# Genome Size Changes by Duplication, Divergence, and Insertion in *Caenorhabditis* Worms

**DOI:** 10.1093/molbev/msad039

**Published:** 2023-02-20

**Authors:** Paula E Adams, Victoria K Eggers, Joshua D Millwood, John M Sutton, Jason Pienaar, Janna L Fierst

**Affiliations:** Department of Biological Sciences, The University of Alabama, Tuscaloosa, AL, USA; Current address: Department of Biological Sciences, Auburn University, Auburn, AL, USA; Department of Biological Sciences, Florida International University, Miami, FL, USA; Department of Biological Sciences, The University of Alabama, Tuscaloosa, AL, USA; Department of Biological Sciences, The University of Alabama, Tuscaloosa, AL, USA; Current address: Absci, Vancouver, WA, USA; Department of Biological Sciences, Florida International University, Miami, FL, USA; Institute of the Environment, Miami, FL, USA; Department of Biological Sciences, Florida International University, Miami, FL, USA; Biomolecular Sciences Institute, Miami, FL, USA

**Keywords:** structural variants, mutation, reproductive transitions, molecular divergence

## Abstract

Genome size has been measurable since the 1940s but we still do not understand genome size variation. *Caenorhabditis* nematodes show strong conservation of chromosome number but vary in genome size between closely related species. Androdioecy, where populations are composed of males and self-fertile hermaphrodites, evolved from outcrossing, female-male dioecy, three times in this group. In *Caenorhabditis*, androdioecious genomes are 10–30% smaller than dioecious species, but in the nematode *Pristionchus*, androdioecy evolved six times and does not correlate with genome size. Previous hypotheses include genome size evolution through: 1) Deletions and “genome shrinkage” in androdioecious species; 2) Transposable element (TE) expansion and DNA loss through large deletions (the “accordion model”); and 3) Differing TE dynamics in androdioecious and dioecious species. We analyzed nematode genomes and found no evidence for these hypotheses. Instead, nematode genome sizes had strong phylogenetic inertia with increases in a few dioecious species, contradicting the “genome shrinkage” hypothesis. TEs did not explain genome size variation with the exception of the DNA transposon *Mutator* which was twice as abundant in dioecious genomes. Across short and long evolutionary distances *Caenorhabditis* genomes evolved through small structural mutations including gene-associated duplications and insertions. Seventy-one protein families had significant, parallel decreases across androdioecious *Caenorhabditis* including genes involved in the sensory system, regulatory proteins and membrane-associated immune responses. Our results suggest that within a dynamic landscape of frequent small rearrangements in *Caenorhabditis*, reproductive mode mediates genome evolution by altering the precise fates of individual genes, proteins, and the phenotypes they underlie.

## Introduction

The evolution of genome size is a fundamental question in biology (Gregory 2005b). Comparative studies in eukaryotes have found genome size variation across large phylogenetic and physical scales is often explained by repeat content (Gregory 2005a; Elliott and Gregory 2015; Kapusta et al. 2017). Here, we ask if these dynamics explain genome size variation at smaller physical and phylogenetic scales in nematodes, a group with defined reproductive transitions and correlated genome size differences.

Nematodes are a compelling system for studying the evolution of genetic and genomic variation. Dioecious *Caenorhabditis* are characterized by molecular “hyper diversity” and little linkage disequilibrium (Cutter et al. 2006; Dey et al. 2013; Crombie et al. 2019; Lee et al. 2021), whereas the androdioecious *C. elegans* has remarkably low levels of genetic variation (Cutter et al. 2009; Noble et al. 2021) and little global population structure (Andersen et al. 2012; Crombie et al. 2019). Despite this there is substantial genome size divergence between *C. elegans* strains. The genome of the “Hawaiian” CB4856 strain contains an extra 4 Mb of genomic sequence when compared with the laboratory standard “Bristol” N2 (Thompson et al. 2015; Kim et al. 2019). Sequencing and analysis across hundreds of wild-collected *C. elegans* strains indicates 2–10% difference in genome size is common (Cook et al. 2017), with copy number (Maydan et al. 2010) and gene presence-absence variation (Lee et al. 2022) contributing to these differences.

Self-fertile *Caenorhabditis* have genomes 10–30% smaller than related outcrossing species (Bird et al. 2005; Haag et al. 2007; Fierst et al. 2015; Kanzaki et al. 2018; Yin et al. 2018), but across the group both the direction of change and the mechanism of genome size evolution remain unanswered questions. Hypotheses include gene deletion and genome reduction in self-fertile species (Thomas et al. 2012; Fierst et al. 2015; Yin et al. 2018) or growth across outcrossing species (Kanzaki et al. 2018; Stevens et al. 2019). Similar patterns of smaller self-fertile genomes and larger outcrossing genomes have been reported in plants including *Arabidopsis* (Hu et al. 2011) and *Capsella* (Slotte et al. 2013) with equivocal support for different mechanisms of expansion and reduction. In the nematode *Pristionchus*, androdioecious species do not consistently have smaller genome sizes when compared with outcrossers in the group (Prabh et al. 2018). Large phylogenetic distances and rapid protein evolution have made it difficult to pinpoint the basis of these genome size changes.

Models of genome evolution often invoke TE-related changes to explain size variation. For example, the “accordion model” proposes genomes grow and shrink by a balance of TE-associated expansions and segmental deletions (Kapusta et al. 2017). Population genetic theory predicts that Class I TEs involving an extrachromosomal RNA intermediate (retroelements) may be differentially affected by the evolution of self-fertility when compared with Class II TEs (DNA elements) with a “cut-and-paste” mechanism (Dolgin and Charlesworth 2006; Boutin et al. 2012). In contrast, *Caenorhabditis* size differences reflect outcrossing species higher gene number and larger protein-coding genome content (Thomas et al. 2012; Fierst et al. 2015; Kanzaki et al. 2018; Yin et al. 2018; Stevens et al. 2019; Teterina et al. 2020; Noble et al. 2021). Technology has limited our ability to address these questions. TEs retain high sequence similarity and these nearly-similar sequences are often collapsed or missing from draft assembled sequences (Ekblom and Wolf 2014; Kim et al. 2019; Blommaert 2020; Pflug et al. 2020; Chen and Zhang 2021). Accurate genomic quantification and characterization are necessary to test theoretical predictions and infer relationships between pattern and process.

Here, we study genome size evolution across a range of divergence times. We test the hypothesis of “genome shrinkage” in self-fertile species (Yin et al. 2018) with a phylogenetic comparative analysis across *Caenorhabditis* and *Pristionchus*. Most assembled genome sequences are still highly fractured, and only a few species have chromosome-scale sequences. We focused our characterization of protein-coding genes and TEs to these well-characterized genomes in the Elegans group of *Caenorhabditis* (Kiontke et al. 2004; Kiontke and Fitch 2005; Kiontke et al. 2011; Stevens et al. 2019; Carlton et al. 2022). Using these highly characterized genomes, we tested the “accordion model” of TE expansion (Kapusta et al. 2017), and Class I/Class II TE dynamics in androdioecious and dioecious species (Dolgin and Charlesworth 2006; Boutin et al. 2012; Makalowski et al. 2019).

We find that nematodes have a strong phylogenetic component to genome size and androdioecious species have not experienced genome reductions. Instead, specific dioecious *Caenorhabditis* lineages have evolved larger genomes through clade-specific combinations of TEs, protein-coding genes and intergenic regions. Elegans group genome evolution is characterized by numerous small-scale rearrangements, even between closely related strains within species. Ancestral genome reconstruction shows that the most common mode of genomic expansion within outcrossing species is the excess accumulation of duplicated sequences. Despite a lack of overall genome size reduction, we find 71 gene families have decreased across self-fertile *Caenorhabditis*. Significant, parallel reductions occurred in self-fertile species’ sensory systems, regulatory proteins and membrane-associated immune responses. These protein changes reflect the shifts in selection that occur with self-fertility including a reduced need to find a mate or deal with pathogens. Overall, our results suggest the evolution of nematode self-fertility results in discrete evolutionary change within a variable, dynamic landscape of genome evolution.

## Results

### Ancestral Genome Size Reconstruction Suggests Androdioecious Genomes Were Not Reduced

**Fig. 1.**
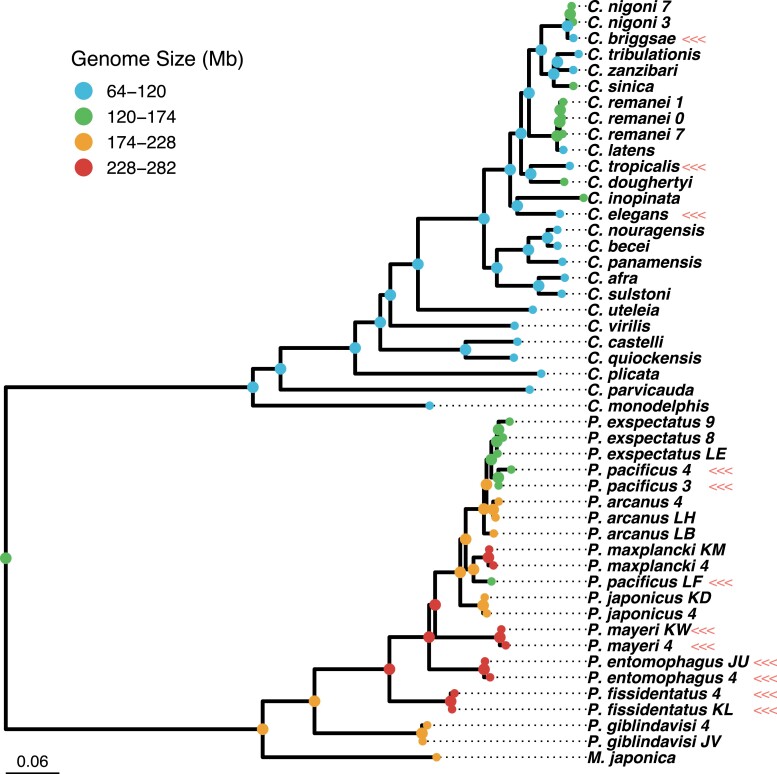
Ancestral state reconstruction shows androdioecious species (marked with triple arrows) do not have reduced genome sizes in either *Caenorhabditis* or *Pristionchus*. Modern genome size estimates were obtained from Stevens et al. (2019), Sutton et al. (2021), and Prabh et al. (2018). Ancestral genome size was estimated with ape (Paradis and Schliep 2018) along the nematode phylogeny estimated with RAxML (Stamatakis 2014) by Ahmed et al. (2022) with branch lengths are in terms of nucleotide substitutions. Additional details are given in the “Ancestral state reconstruction and phylogenetic comparative analysis of genome size” section of the Materials and Methods.

We examined patterns of genome size evolution by assigning “selfer” and “outcrosser” states to the tip species of a phylogeny containing *Caenorhabditis* and *Pristionchus* species with *Parapristionchus* and *Micoletzkya* included as dioecious outgroups (Ahmed et al. 2022). We then reconstructed ancestral genome sizes from measured extant species genome sizes (Prabh et al. 2018; Stevens et al. 2019; Sutton et al. 2021; supplementary fig. S1, Supplementary Material online) using a Brownian motion model of evolution and REML to estimate likely ancestral node genome sizes and 95% confidence intervals (Paradis and Schliep 2018; supplementary table S1, Supplementary Material online). We did not discern a reduction in androdioecious species genome sizes relative to dioecious species through time. Instead, the entire *Caenorhabditis* clade underwent a genome size reduction early on in their evolution, and the three extant androdioecious species retained this reduced state (fig. 1). A few dioecious *Caenorhabditis* appear to have increased genome size and all of these form groups of closely related species or strains that are sister to the androdioecious species. Comparing extant dioecious species to extant androdioecious *Caenorhabditis* without taking the broader phylogenetic context into account would lead to the conclusion that the latter have undergone genome size reductions. Re-enforcing this point, in *Pristionchus* three of the androdioecious samples retained the estimated ancestral genome size, whereas six actually exhibited some of the largest genome sizes amongst extant species (fig. 1).

### TE Content Was Not Predicted by Reproductive Mode

We sought to identify the basis of genome size evolution by testing hypotheses of TE-associated change. Draft assembled genome sequences are available for the *Caenorhabditis*, *Pristionchus*, *Parapristionchus* and *Micoletzkya* used in ancestral state reconstruction but many of these remain fractured and incomplete (Stevens et al. 2019). In order to rigorously test TE-associated hypotheses we focused on a high-confidence set of species from the Elegans group with chromosome-scale assembled sequences (supplementary table S2, Supplementary Material online) across a range of evolutionary divergence scales. We started very small with a comparison between the genomes of three strains of the outcrossing nematode *C. remanei*. We included *C. latens*, a dioecious species originally classified as a strain of *C. remanei* and later defined as a separate species (Dey et al. 2012). We then broadened our comparisons to include the outcrossing *C. nigoni*, *C. inopinata*, and *C. sinica* and the self-fertile *C. elegans*, *C. briggsae*, and *C. tropicalis*, all members of the Elegans group representing over 100 million years of divergence.

**Fig. 2.**
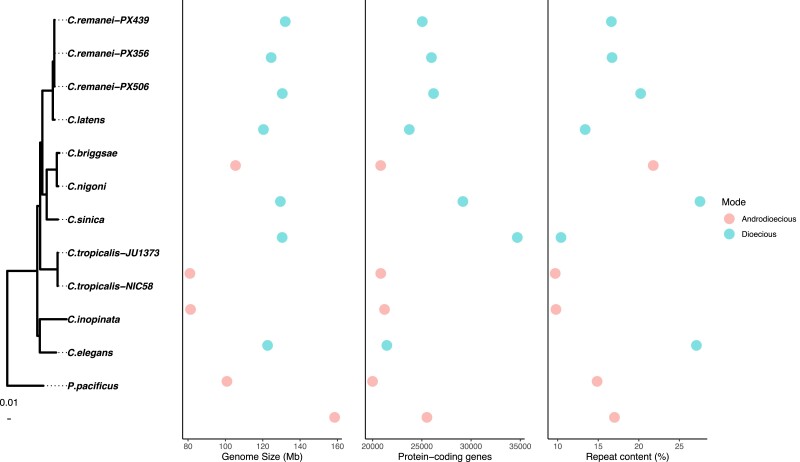
Genome size and number of protein-coding genes varied with reproductive mode in the Elegans group whereas repeat content did not. The *Caenorhabditis* phylogeny was estimated with RAxML (Stamatakis 2014) by Ahmed et al. (2022) with *P. pacificus* used as an outgroup to root analyses. Branch lengths are in terms of nucleotide substitutions. Genome size, number of protein coding genes, and repeat content were annotated as described in the “Gene annotation” and “TE annotation” sections of the Materials and Methods and measured with AGAT stats (Dainat).

Despite consistent differences in genome size between outcrossing and self-fertile species, there were no consistent differences in repeat content (fig. 2). Genome size variation within self-fertile and outcrossing species also did not reflect differences in repeat content. For example, *C. inopinata* had one of the smallest outcrossing genomes at 122 Mb but one of the highest repeat contents at 27.1% (fig. 2). Outcrossing genomes varied between 10.41% and 27.54% repetitive content and self-fertile genomes varied between 9.7% and 21.78% repetitive content.

Class I and Class II TE expansion has been hypothesized to vary with reproductive mode (Dolgin and Charlesworth 2006; Boutin et al. 2012). In the Elegans group Class II TEs varied similarly across reproductive modes with ranges of 6.96–19.63% in outcrossing genomes and 6.13–16.38% in self-fertile genomes. However, outcrossing genomes had higher proportions of Class I TEs (0.4–4.85%) when compared with self-fertile species (0.68–1%).

To statistically evaluate Class I TE differences and the “accordion model” of TE expansion whilst accounting for potential pseudoreplication due to common ancestry, we performed a phylogenetic comparative analysis. We first mapped discrete “selfer” or “outcrosser” states onto the phylogeny by distinguishing “equal rates,” “symmetrical” and “all rates different” transition matrix models using the small sample size corrected Akaike Information Criteria (AICc). We found “symmetrical” and “equal” rate transition matrix models both out-performed the “all rates different” model, but were indistinguishable from each other by AICc (table 1). We discretized ancestral branch states by choosing the state with the highest probability for each internal branch and used these as hypotheses for how long the different lineages evolved as selfers or outcrossers to parameterize separate optima Ornstein–Uhlenbeck models for each TE. In all but one case, either the Brownian Motion or the Ornstein–Uhlenbeck global optima model performed best, outperforming the Ornstein–Uhlenbeck separate optima model by more than two AICc units (the criteria for significantly different suggested by Burnham and Anderson 2002).

**Table 1.** Specific TE Elements and the AICc Scores for a Brownian Motion (BM), Single Optimum Ornstein–Uhlenbeck Model (OU1) and Separate Self-fertile and Outcrossing Optima Ornstein–Uhlenbeck Model (OU2).

Element		AICc (BM)	AICc (OU1)	AICc (OU2)
Repeats (total)		73.83	74.86	77.94
GC %		24.33	28.26	27.59
Retroelements		38.06	40.64	43.84
	Penelope	−51.02	−47.09	−43.06
	LINEs	1.49	1.59	6.61
	L2 CR1 Rex	−12.83	−14.25	−10.29
	R2 R4 NeSL	−37.05	−42.28	−37.49
	RTE Bov B	−17.74	−13.81	−9.21
LTR elements		34.49	37.32	39.91
	BEL Pao	−19.89	−19.86	−15.10
	Gypsy	20.76	24.48	27.45
DNA transposons		57.25	61.17	64.09
	hobo Activator	−1.73	2.20	7.03
	TC1 IS630 Pogo	42.08	46.01	50.93
	PiggyBac	−5.95	−2.04	0.37
	Mutator	51.41	51.00	49.21
Other (Mirage, P elements, Transib)		62.05	58.63	62.87
Helitrons/rolling circles		15.93	19.86	25.10
Unclassified repeats		72.05	42.61	45.13
Total interspersed repeats		72.78	73.52	75.91
Satellites		12.51	16.44	21.66
Simple repeats		−25.85	−21.92	−23.05

Note.—Underlined AICc values indicated either the single best model or in some cases the best model and next best model that could not be distinguished by more than 2 AICc units. Eighteen of the 21 TEs were best modeled by a BM process and four of these could not be distinguished from a single optimum OU process. Five TEs were best modeled as a single optima OU process, two of which could not be distinguished from a BM model. Only one of the 21 TEs (the DNA transposon Mutator) was best modeled by a separate optimum OU model. The phylogenetic half-life and stationary variance for Mutator were 0.0011 and 1.35 respectively with greater than 60% of the variance explained by the two optima model ($r^{2}=0.64$).

The Ornstein–Uhlenbeck model with separate optima for self-fertile and outcrossing species performed best for the DNA transposon *Mutator* by 1.79 AICc units compared with the next best single optima model. The estimates of primary optima for self-fertile species (2.97% ± 0.58%) and outcrossing species (6.19% ± 0.44%) were greater than two standard errors different from each other and given the low level of phylogenetic inertia estimated ($t_{1/2}=0.11\text{\%}$ of the total tree height), were similar to the currently observed mean values within self-fertile and outcrossing species. *Mutator* elements in self-fertile genomes ranged from 5,234 in *C. briggsae* to 12,076 in *C. tropicalis* JU1373. In comparison, *Mutator* elements in outcrossing genomes ranged from 24,803 in *C. nigoni* to 47,268 in *C. inopinata*. However, we note that this DNA transposon makes up 1–8.15% of the respective genomes and *Mutator* alone cannot explain the much larger differences in overall genome size (12–66%).

### Variation in Protein-Coding Gene Number

As previously reported, the number of protein-coding genes in Elegans varied with reproductive mode (Thomas et al. 2012; Fierst et al. 2015; Stevens et al. 2019; Teterina et al. 2020; Noble et al. 2021). Outcrossing species contained 21,443–34,696 genes whereas self-fertile species contained 19,997–21,210 genes (fig. 2). The fragmented *C. sinica* sequence (supplementary table S2, Supplementary Material online) may have an artificially inflated estimated gene number of 34,696. Excluding *C. sinica* the mean number of genes in outcrossing genomes was 25,258 as compared with 20,714 in self-fertile genomes.

The higher number of protein-coding genes in outcrossing species resulted in an overall larger genic footprint (supplementary tables S3 and S4, Supplementary Material online). The total portion of the genome dedicated to producing mRNA ranged from 63.6–68.12 Mb in outcrossing species and 48.86–63.98 Mb in self-fertile species. The exception was the outcrossing *C. remanei* PX506 which has only 56.4 Mb of mRNA. Computational predictions of protein-coding genes are, at best, 75–85% accurate (Bruna et al. 2021) and some of these differences likely reflect annotation false positives and negatives. Beyond protein-coding genes and repeats, unannotated intergenic regions comprised 15.81–37.04% of Elegans genomes (supplementary tables S3 and S4, Supplementary Material online).

### Elegans Genome Evolution Was Characterized by Extensive Gene-Associated Duplicated and Diverged/Inserted Regions

**Fig. 3.**
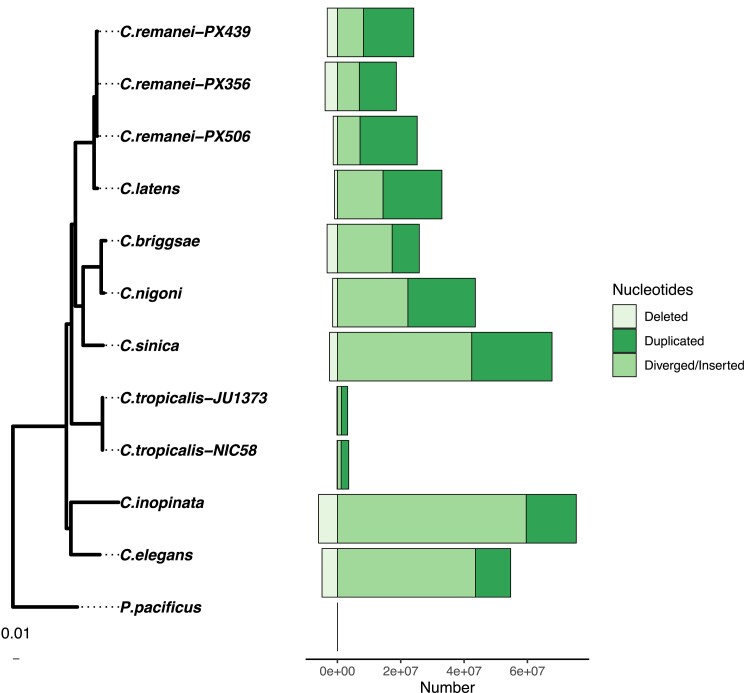
*Caenorhabditis* genome evolution is characterized by duplications and insertions with infrequent deletions. For most species insertions map to gene regions with the exception of *C. remanei* PX506. Genome changes were measured relative to the last common ancestor between each pair of species. The x-axis denotes the direction of genome size change with negative values regions that were lost through deletion and positive values regions that were gained relative to the reconstructed ancestral genome. As in fig. 2, the *Caenorhabditis* phylogeny was estimated with RAxML (Stamatakis 2014) by Ahmed et al. (2022) with branch lengths in terms of nucleotide substitutions.

Genomic changes from ancestor to child can occur in multiple ways (supplementary fig. S3, Supplementary Material online). We aligned Elegans genomes, reconstructed ancestral sequences and defined mutations between ancestors and descendants. Deletions represented a small fraction of observed changes between both closely and more distantly related species pairs. The most common nucleotide changes across the Elegans group were duplications and insertions (fig. 3). Rapid nucleotide divergence could potentially result in an overrepresentation of “inserted” sequences. Highly divergent nucleotide regions could mutate to a point where pairwise alignment was impossible, resulting in false positive “inserted” sequences. For example, in *C. elegans* “hyper-divergent” haplotypes constitute 20% of the genome (Lee et al. 2021). We call these “Diverged/Inserted” sequences to acknowledge this possibility.

For closely related species like the *C. remanei*/*C. latens* species group, mutations can be tracked at a high resolution. For example, for *C. remanei* PX506 we find that 1.33 Mb of sequence was deleted relative to the ancestor (1% of the *C. remanei* PX506 genome size), 2% of nucleotides changed through substitution, 5% of the nucleotides were inserted/diverged and 14% of the nucleotides were duplicated (fig. 3). In comparison, 92% of the nucleotides were aligned/matched to the reconstructed ancestor sequence. Nucleotide changes can be encased in larger genomic rearrangements and sum to a representation greater than the current genome size (i.e., greater than 100%). Despite this, we can compare the relative contribution of each type of genomic change to genome size in the dataset and reject deletions as the predominant mode of genome evolution (df=21, t=4.75, $P=5.47\times 10^{-5}$).

**Fig. 4.**
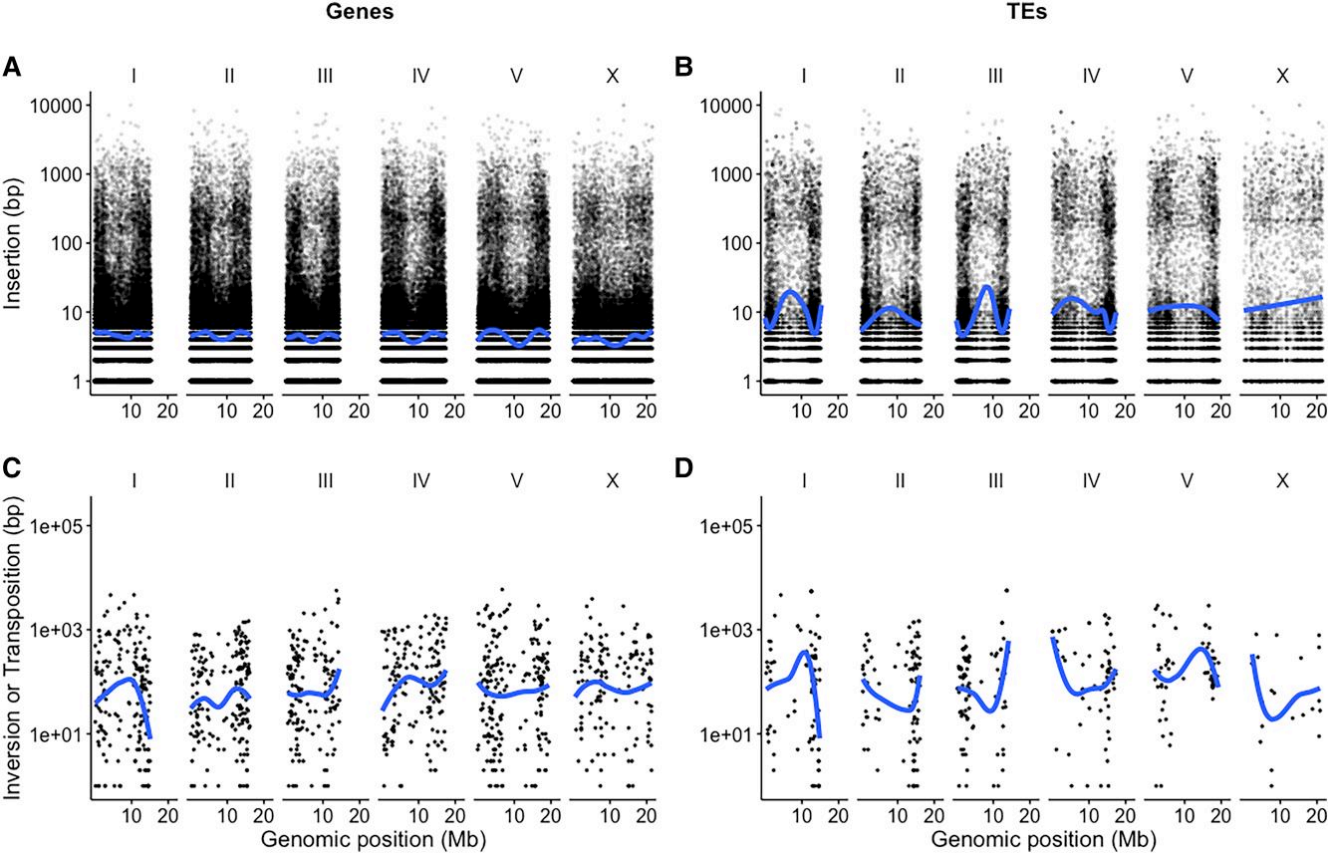
The most frequent type of genomic changes between *C. briggsae* and its ancestor were small gene-associated insertions. Insertions overlapping (*A*) Genes and (*B*) TEs were more frequent and of smaller size when compared with inversions and transpositions overlapping (*C*) Genes and (*D*) TEs. Each point represents one mutation with Chromosome and Genomic position (in Mb) on the *y*-axis and size (in bp) on the *x*-axis. The blue line is the median value estimated across each chromosome with a loess function for plotting.

We associated mutations with genomic features and found that most changes in the Elegans group occurred within genic sequences or unannotated regions (supplementary table S5, Supplementary Material online). For example, in *C. briggsae* 21.78% of the genome contains repeats but only 2% of the total insertions occurred within repeat sequences (fig. 4; df=10, t=2.46, P=0.017). The notable exception to this was in the *C. remanei* group where 28–72% of inserted sequences were located in TE regions whereas only 16.6–20.2% of these genomes were annotated repeats. Changes within and close to TEs were less frequent but of larger mean and median size in all genomes (supplementary table S6, Supplementary Material online). Inversions and transpositions occurred 1–2 orders of magnitude less frequently than Diverged/Inserted regions.

**Fig. 5.**
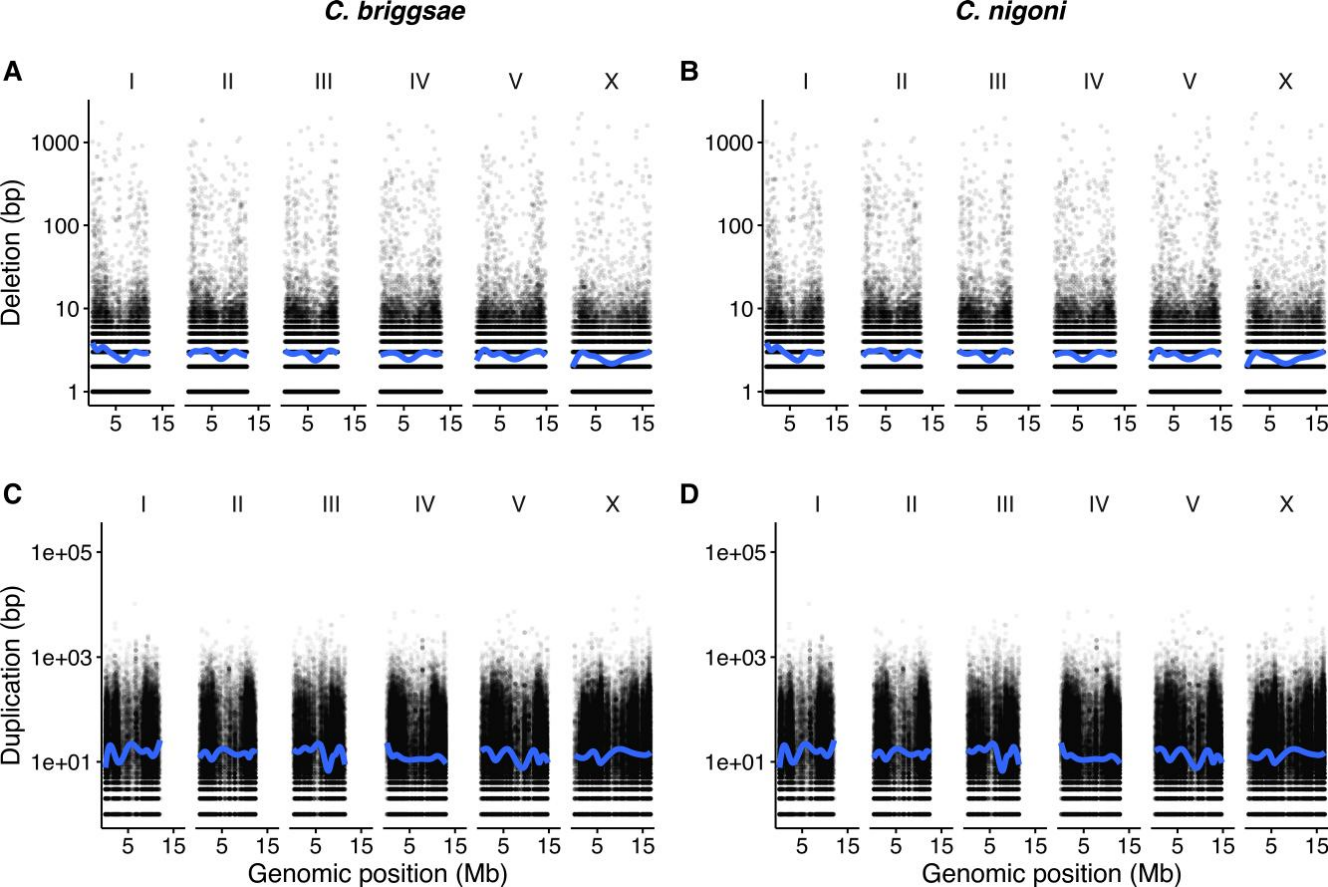
The genomes *C. nigoni*, the most closely related outcrossing species to *C. briggsae*, experienced similar mean and median sizes of Deletions and Duplications. Each point represents one mutation with Chromosome and Genomic position (in Mb) on the y-axis and size (in bp) on the x-axis. The blue line is the median value with a loess function for plotting.

Duplications were more frequent than deletions and had larger mean and median sizes (supplementary table S7, Supplementary Material online). Deletion and duplication patterns were similar for both self-fertile and outcrossing Elegans. For example, the mean and median sizes of deletions and duplications were similar for both the self-fertile *C. briggsae* and the outcrossing *C. nigoni* (fig. 5). Insertions, inversions and tranpositions were calculated in the species genome coordinates whereas deletions and duplications were calculated in the ancestral genome coordinates. This prevented us from inferring overlap with genomic features as ancestral genomes were not reconstructed at a sufficiently high resolution to annotate protein-coding genes and TEs.

### Gene Family Turnover Was High Across Elegans

**Fig. 6.**
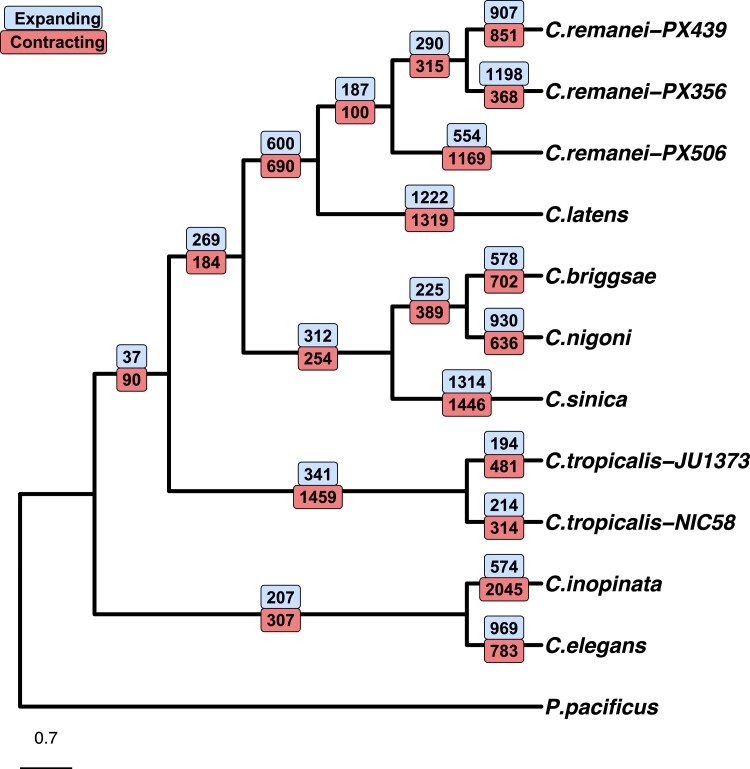
The number of gene family expansions and contractions on each branch of the *Caenorhabditis elegans* phylogeny as estimated by CAFE5 (Mendes et al. 2020). A detailed description of the analyses are given in the Materials and Methods section “Gene birth and death analyses.” The *Caenorhabditis* phylogeny was estimated with RAxML (Stamatakis 2014) by Ahmed et al. (2022) and branch lengths are in terms of nucleotide substitutions.

We used OrthoFinder (Emms and Kelly 2015) to associate protein-coding genes to orthogroups or gene families and estimated gene birth and death for 14,590 high-confidence orthogroups with the CAFE5 software (Hahn et al. 2005; Han et al. 2013; Mendes et al. 2020). We tested models with 1–9 different birth/death rates (λs) for gene family change and found that a model with 2 birth/death rates (λ) had the highest likelihood. The estimated birth/death rate across the Elegans group (λ=0.4293) was 1–2.5 orders of magnitude higher than that reported in *Drosophila* (Hahn et al. 2007) or *Saccharomyces* (Han et al. 2013). Rapid gene family expansion and contraction was not limited to one clade or individual species (fig. 6).

As an illustration of this we studied F-box FBA2 (*fbx-a*) genes in the *C. elegans*-*C. inopinata* selfing-outcrossing species pair. F-box genes are involved in protein-protein interactions (Kipreos and Pagano 2000), and implicated in the sexual system in *Caenorhabditis*. For example, recruitment of an F-box gene to a pathway regulating *C. elegans* hermaphrodite development was a crucial piece of evidence for the three independent origins of self-fertility in Elegans (Guo et al. 2009). F-box genes occur in Elegans genomes in high numbers with at least 377 in *C. elegans* (Wang et al. 2021). Of these, 222 are annotated as F-box FBA2 (*fbx-a*) genes characterized by an F-box domain and an FBA2 domain. The proteins are unevenly spread across the chromosomes with 6 on chromosome IV in *C. elegans* and 92 on chromosome V.

**Fig. 7.**
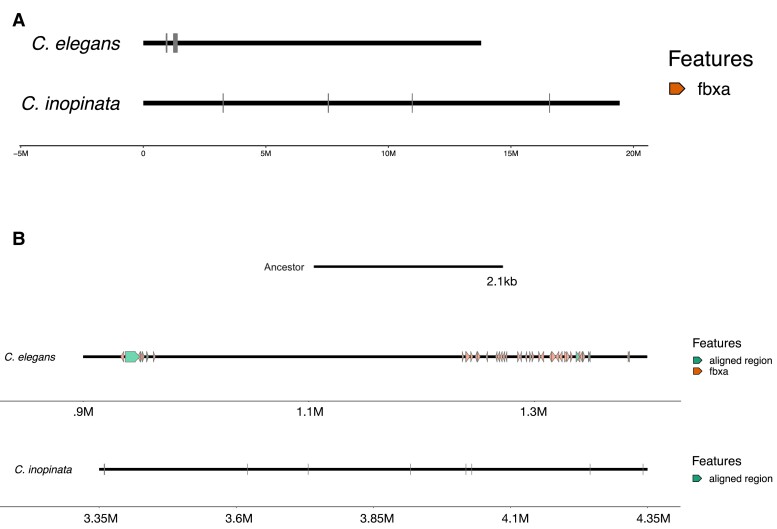
(*A*) A 500 kb region of *C. elegans* chromosome III contained 46 annotated fbx-a genes. In comparison there are 4 fbx-a genes spread across 15 Mb of chromosome III in *C. inopinata*. (*B*) A 2.1 kb region of ancestral sequence aligned to this region multiple times in *C. elegans* with high sequence similarity but aligned weakly to small, disjunct regions in *C. inopinata*.

We identified a 2.1 kb region of ancestral sequence (fig. 7) that aligned to multiple regions within a 500 kb section of *C. elegans* chromosome III (identified as duplicated sequence). The same region of ancestral sequence was not identified in the *C. inopinata* genome and instead aligned poorly to small, disjunct regions across the chromosome. Biological function is not known for F-box FBA genes but they appear to have arisen in high numbers in *Caenorhabditis* through tandem duplications (Wang et al. 2021) with population genetic signatures of strong positive selection (Ma et al. 2021). F-box and F-box FBA genes are abundant in outcrossing *Caenorhabditis* as well, with 1,358 annotated F-box proteins and 412 annotated *fbx-a* genes in the outcrossing *C. remanei* PX506. Rapid gene birth and death via small structural mutations were common in both outcrossing and self-fertile Elegans worms.

### Protein Families Show Parallel Decreases Across Self-Fertile Species

We found that of the 14,590 gene families 564 were expanding or contracting significantly at q<0.01 (supplementary fig. S13, Supplementary Material online). Of these, 71 were decreasing in parallel across self-fertile species relative to outcrossing species. We identified an annotated protein domain or *C. elegans* orthologous protein for genes in 30 of these families. Five families included serpentine receptors, chemoreceptors known to be important for a worm’s ability to navigate its environment. Three of the families encoded F-box associated genes and 10 of the families encoded regulatory proteins including DNA polymerase-associated domains, RNA export domains, histone-lysine N-methyltransferases, kinases and zinc finger motifs like Broad-Complex, Tramtrack, Bric a brac (BTB) and RING. We identified eight families encoding membrane associated immune and protein responses including C-type lectins, ankyrin repeats, actin and chitin associated proteins and peptidases including the Chymotrypsin family.

*Caenorhabitis* genomes contained large numbers of each of these protein families and changes in gene number could reflect overall changes at the genomic level. F-box associated proteins and kinases were not changing significantly (P=0.15 and P=0.14, respectively) whereas serpentine receptors (P<0.001), transcription factors (P=0.0001) and peptidases were (P=0.0026). Thus, gene family expansions and contractions likely reflected a mix of true selection and drift mediated by high molecular turnover. We did not identify gene families consistently increasing in parallel in selfing species or increasing or decreasing in outcrossing species.

## Discussion

We have presented a comparative analysis of genome size evolution in androdioecious and dioecious nematodes, and a fine-scaled analysis of genome evolution across the Elegans group. We tested hypotheses proposing different sources of genome size variation between outcrossing and self-fertile species including “genome shrinkage” (Yin et al. 2018), the “accordion” model of TE expansion and segmental deletion (Kapusta et al. 2017), and differences in Class I and Class II TE dynamics (Dolgin and Charlesworth 2006; Boutin et al. 2012). We found no evidence for these hypotheses as the basis of genome size differences in these species. Instead, our results show genome evolution in Elegans is characterized by high rates of small structural mutations, particularly duplications and insertions, coupled with rapid nucleotide divergence.

### *Caenorhabditis* Genomes: Dynamic Evolutionary Change

The dominant hypothesis for genome evolution after the advent of self-fertility has been deletion of genes (Thomas et al. 2012; Fierst et al. 2015; Rodelsperger et al. 2018; Yin et al. 2018) and loss of DNA (Hu et al. 2011; Shimizu and Tsuchimatsu 2015; Roessler et al. 2019). We found no evidence for this in *Caenorhabditis* or *Pristionchus*. Our results suggest instead that specific dioecious *Caenorhabditis* genomes expanded via numerous duplications and insertions coupled with rapid nucleotide divergence.

These findings agree with a large-scale study describing the genomes of 10 newly discovered *Caenorhabditis*. Stevens et al. (2019) reported a substantial phylogenetic influence on genome size, with the *Drosophilae* Super-group (the clade at the base of the Elegans group) having small genome sizes 65–91 Mb and protein-coding gene counts dipping to just 17,134. Notably, all of these species are outcrossing. The lower bound of these estimates is roughly half that observed for both genome size and gene count in outcrossing Elegans *Caenorhabditis* and these two clades comprise the upper and lower bounds for estimates of genome size and gene count across the genus. These results suggest that reproductive transitions and the associated influences on genome evolution in *Caenorhabditis* occur against a background of dynamic genome evolution characterized by frequent SV mutations and genome size changes.

We found that *Caenorhabditis* had high rates of gene family turnover including both birth and death rates, and resulting expansions and contractions across the phylogeny. These rates were 1–2.5 orders of magnitude higher than those previously reported for other metazoans (Hahn et al. 2005, 2007; Han et al. 2013; Schrader et al. 2021). Although SV mutations are randomly generated, they appear to be contributing to rapid rates of gene duplication and in some instances deletion (He et al. 2019; Kim et al. 2019). These high rates have been experimentally observed in *C. elegans* (Lipinski et al. 2011; Konrad et al. 2018), to the point that duplications and deletions arise in parallel in replicate populations (Farslow et al. 2015). Mutation accumulation studies focusing on the self-fertile *C. elegans* report duplications occurring at an estimated $2.9\times 10^{-5}$/gene per generation (Lipinski et al. 2011; Katju and Bergthorsson 2013; Farslow et al. 2015) and deletions at $5\times 10^{-6}$/gene per generation (Konrad et al. 2018). In comparison, single nucleotide base substitutions occur at $10^{-9}$ to $10^{-8}$ per generation (Denver et al. 2004).

Given this rich mutational spectrum, it can be challenging to infer which changes occur as a consequence of reproductive mode. We found no gene families consistently expanding or contracting in outcrossing species, or expanding in self-fertile species. Of the 564 gene families that were significantly changing in Elegans, 71 (12.6%) were contracting in parallel across self-fertile genomes. A transcriptome-based study in *Pristionchus* noted hermaphroditic lineages lost BTB domain-containing proteins, C-type lectins and chymotrypsin domains (Rodelsperger et al. 2018). Importantly, our analyses indicated these have also been lost in self-fertile *Caenorhabditis*. The evolution of self-fertility produces similar selective pressures across nematodes and the loss of similar genes suggests parallel protein responses across diverse lineages. Little is known about the function of these broad categories in nematodes and these gene families are compelling candidates for further study regarding the selection pressures accompanying androdioecy.

The average *Caenorhabditis* gene size is just 3 kb, and these gene reductions contribute little to overall genome size differences. However, individual genes and proteins can be extremely significant for organismal evolution. An important example of this is the *male secreted short*, or *mss*, protein family that is required for sperm competition in outcrossing species and has been largely lost in self-fertile *Caenorhabditis* (Yin et al. 2018; Yin and Haag 2019). *Caenorhabditis* have an extremely short mutational path from dioecious outcrossing to self-fertility (Baldi et al. 2009), but the transition to androdioecy puts the population into an entirely different selection regime. An outcrossing organism must be able to navigate its environment to find the opposite sex, successfully mate, and frequently deal with many more pathogens and parasites (Andersson 1994). In multiply-mating species these behavioral and environmental demands exert strong selective pressures on males (Shuster and Wade 2003) and, accordingly, male-biased and male-associated genes are preferentially missing in both androdioecious *Caenorhabditis* (Thomas et al. 2012; Yin et al. 2018) and *Pristionchus* (Rodelsperger et al. 2018). Our results suggest that similar patterns are occurring in proteins responsible for sensory recognition, regulatory systems, and membrane-associated immune and protein responses. These genomic changes reflect the comprehensive environmental selection pressures that mating systems impose. The transition to androdioecy also demands that populations survive substantial inbreeding depression (Dolgin et al. 2007) by fixing specific haplotypes and epistatically interacting loci (Adams et al. 2022). Thus, although the overall size dynamics were not explained by differential loss of genes or DNA, precise protein losses and genomic alterations have been mediated by the evolution of self-fertility.

### Genome Size Is Not Predicted by TE Content

We found the observed differences in *Caenorhabditis* genome size were not due to TEs. There was no evidence for differences in overall TE content, differential expansion and deletion (the accordion model) (Kapusta et al. 2017) or dynamics of Class I/Class II TEs (Dolgin and Charlesworth 2006; Boutin et al. 2012). Our results add to studies showing little change in TE abundance after the evolution of self-fertility (Hu et al. 2011; Slotte et al. 2013) and little difference in the evolutionary dynamics of Class I and Class II TEs (Nowell et al. 2021). However, this absence of evidence is not conclusive evidence against these hypotheses. Large regions of most assembled genomes remain uncharacterized and some 10–40% of Elegans genomes are unannotated intergenic regions. A large portion of the story of Elegans genome evolution lies in this uncharacterized “dark matter” (Mefford 2014), and will remain elusive until we can better identify both TEs and protein-coding sequences.

Models addressing reproductive systems and their influence on TE dynamics make specific predictions based on the relative age of the sexual system and TE invasion into the genome (i.e., recent vs ancient) and are sensitive to variation in parameters like quantitative rates of outcrossing and transposition (Boutin et al. 2012). These subtle variations may act in populations but not be discernible in broad-scale comparative analyses across multiple types of TEs and long evolutionary divergence times. In the future, fine-scale studies that are better able to match experimental data with theoretical parameters may result in greater insights into the interactions between reproductive systems and TE evolution.

The one exception to this general lack of differentiation in TEs was the DNA transposon *Mutator*. *MUtator*-Like Elements (MULEs) draw their name from their mutagenic abilities as the TEs are highly active near genes and frequently acquire host gene fragments (Dupeyron et al. 2019). Our phylogenetic comparative analysis found the outcrossing primary optimum was roughly twice that of the self-fertile primary optimum for *Mutator* TEs, which results in two interesting biological questions. First, why is the *Mutator* content higher in outcrossing species? And second, are Mutator dynamics responsible for the high rates of rearrangements we observed in *Caenorhabditis* genomes?

*Mutator* transposons were first discovered in maize where it was found that lines with the TE had mutation rates 30× higher than those without (Robertson 1978). Since then *Mutator* transposons and MULEs have been extensively studied in maize and used as forward and reverse mutagenesis systems (Lisch 2015). In addition to high rates of transposition activity, Pack-*MUtator*-Like transposable Elements (Pack-MULEs) modify genes through biased acquisition of GC-rich sequences and preferential insertion near the $5^{\prime }$ end of transcripts (Jiang et al. 2011). *Mutator*-based mutagenesis overwhelmingly affects genes because of these unique sequence-level mechanisms. Multiple generations of self-fertilization can silence *Mutator* transposons in maize (Robertson 1986) through DNA methylation (Chandler and Walbot 1986; Martienssen and Baron 1994; Slotkin 2005) that heritably modifies histones (Guo et al. 2021).

These results in maize suggest that *Mutator* transposons may be differentially affected by outcrossing and self-fertilization, and the contribution to mutational dynamics in maize may also occur in nematodes. However, *Mutator* transposons have only recently been discovered in metazoans and there is little known about how their dynamics may be similar to or differ from those observed in plants (Liu and Wessler 2017). For example, despite the interesting parallel with self-fertilization DNA methylation is absent in *Caenorhabditis* (Simpson et al. 1986; Wenzel et al. 2011) and any mechanism of *Mutator* control in worms would have to occur through a separate mechanism. *Mutator* transposons in nematodes, particularly *Caenorhabditis*, may be an exciting avenue for future studies of mutational dynamics.

Until recently, the technology used to assemble and characterize genome sequences has had an outsize influence on the inference of genome size, protein-coding genes, and TEs and our ability to test theoretical predictions regarding genome evolution. For example, a prominent hypothesis linking large effective population sizes with small genome sizes (Lynch and Conery 2003) generated predictions and explanatory theory for a pattern *opposite* to that observed in worms, plants, and other self-fertile/outcrossing species. As the field of evolutionary biology shifts to newer long read DNA technologies we will be increasingly able to quantify patterns of genome evolution as they apply to phylogenetic groups, reproductive modes, and functional systems like chromosome pairing and meiosis. The ambitious Darwin Tree of Life programme at the Wellcome Sanger Institute is aiming to sequence the 70,000 eukaryotic species in the UK and Ireland with long-read technology and permit high-quality, chromosome-scale assemblies (Blaxter et al. 2022). These resources will be critical for definitively rejecting and critically evaluating hypotheses regarding genome evolution across eukaryotic life.

## Materials and Methods

### Ancestral State Reconstruction and Phylogenetic Comparative Analysis of Genome Size

We obtained *Caenorhabditis* genome size estimates from Stevens et al. (2019) and Sutton et al. (2021), and *Pristionchus*, *Parapristionchus* and *Micoletzkya* genome size estimates from Prabh et al. (2018). To visually examine patterns of genome evolution through time, we reconstructed genome size as a continuous trait using the ape R package (Paradis and Schliep 2018). We used the nematode phylogeny estimated with RAxML (Stamatakis 2014) by Ahmed et al. (2022) as the basis for the reconstruction and added the strains *C. remanei* PX356, PX439, PX506 and *C. tropicalis* JU1373 and NIC58 as polytomies. There is some genetic differentiation between strains within each species but the scale of protein divergence at loci conserved across Phylum Nematoda is minimal within strains when compared with the entire phylum (Bird et al. 2005). Additionally, the inbreeding process necessary to create homozygous strains can lead to unusual fixations and genetic sampling effects that do not accurately represent the species (Roessler et al. 2019; Adams et al. 2022). A Brownian motion process was used to model trait evolution and the most likely ancestral node states were estimated using restricted maximum likelihood (REML).

Hansen (1997) introduced the use of an Ornstein–Uhlenbeck model to test hypotheses of trait adaptation to different niches mapped on a phylogeny. The Ornstein–Uhlenbeck process includes parameters that capture deterministic movement of species trait values toward optimal states that can vary as a function of environmental or ecological variables. Hansen (1997) termed these states “primary optima” defined as the average expected trait values (the local optima) for many species adapting to a given primary niche. The idea is that “secondary” selective factors average out across species, leaving the common effect of the primary niche on trait values. Hypotheses about adaptation can be tested by estimating the primary optima for different states of the environmental or ecological variables and asking if they differ as predicted by the hypotheses (see Hansen 2014 for a detailed argument).

Mathematically, a simple Ornstein–Uhlenbeck process is described by the stochastic differential equation:


(1) dy=−α(y−θ)dt+σdW,

where dy is the change in a given species’ mean trait value, y, over a short time interval dt, θ is the primary optimum, α determines the rate of adaptation toward the primary optimum, dW represents independent normally distributed stochastic changes with mean zero and unit variance over a unit of time, and σ is the standard deviation of these changes. The σ parameter is more readily interpretable when expressed as a stationary variance of the process, $v=\sigma^{2}/2\alpha$, which is the variance among species within a niche after a long period of independent evolution.

Using SLOUCH (Hansen et al. 2008; Kopperud et al. 2018), one of many R packages that implement the methods mentioned above, we investigated whether nematodes sharing a given niche (here, dioecious or androdioecious reproduction) tend to be more similar compared with taxa sharing a different niche in terms of genome size. We simultaneously estimated and controlled for the levels of adaptation and phylogenetic inertia in the clade (see Hansen 2014; Mahler and Ingram 2014 and O’Meara and Beaulieu 2014 for general reviews). A Brownian motion model and two Ornstein–Uhlenbeck models, one with a single global optimum and one with separate primary optima for selfers and outcrossers were tested as hypothesers for genome size evolution. To define ancestral niches, we mapped “selfer” and “outcrosser” as discrete states onto the nematode phylogeny estimated by Ahmed et al. (2022) using maximum likelihood in the Ape R package (Paradis and Schliep 2018). The ancestral states along each branch were discretized by choosing the state with the highest probability on each branch for the best model.

### Assembling Chromosome-Scale Sequences for the Outcrossing *C. Remanei* PX356 and PX439 and *C. Latens*

We used Oxford Nanopore Technologies to generate DNA libraries for *C. remanei* PX356, PX439 and *C. latens* PX534 (Sutton et al. 2021). The two species are closely related (Felix et al. 2014) and partially interfertile (Dey et al. 2014). We assembled genome sequences in <100 contiguous sequences for each strain and used the contiguous *C. remanei* PX506 assembled sequence (Teterina et al. 2020) to scaffold the genome sequences into chromosome-scale pseudomolecules (supplementary table S1, Supplementary Material online).

### Nematode Laboratory Culture

The *C. remanei* PX356 and PX439 and *C. latens* PX534 strains were graciously provided by the lab of Patrick C. Phillips. Nematodes were cultured on 100 mm Nematode Growth Media (NGM) plates seeded with *E. coli* OP50 (Stiernagle 2006). For sequencing we collected worms from 5 to 10 100 mm plates by washing with M9 media into 15 ml conical tubes. Tubes were placed on a tabletop rocker for 1 h and then centrifuged to pellet nematodes. To minimize E. coli contamination we removed the M9, added fresh M9, mixed the tubes and pelleted the worms by centrifugation, repeating this process five times.

### DNA Extraction and Sequencing

Our protocol for DNA extraction was described in Sutton et al. (2021). Briefly, we froze pelleted worms in liquid nitrogen to rupture cuticles and combined 1.2 ml lysis buffer (100 mM EDTA, 50 mM Tris, 1% SDS) with 20 μl Proteinase K (100 mg/ml) before incubating at 56 ${}^{\circ }$C for 30 min with shaking. We used phenol chloroform for extraction following Sambrook (2001) and the Short Read Eliminator Kit from Circulomics Inc. (Baltimore, MD) to select high molecular weight DNA.

We used the Oxford Nanopore Technologies (Oxford, UK) SQK-LSK109 ligation sequencing kit for DNA library preparation. Approximately 500–900 ng of DNA was sequenced for 48 h on R9.4.1 RevD fowcells via a gridION X5. We used Guppy v.4.0.11 for basecalling in the “-high-accuracy” mode.

### Genome Assembly Strategy

We used the Canu v2.0 software package to correct Nanopore libraries (Koren et al. 2017). Briefly, Canu’s correction module creates an all-vs-all overlap dataset, uses this to correct individual reads, selects the longest available reads and creates a dataset of user-specified coverage. For the data presented here we used 40× coverage based on an estimated genome size of 130 Mb. We assembled the Canu-corrected reads with Flye v.2.8.2 (Kolmogorov et al. 2019) and polished the assembled sequences with Pilon v1.23 (Walker et al. 2014). We designed this correction, assembly and polishing protocol after extensive simulations and testing (described in Sutton et al. 2021). For polishing we used paired-end Illumina libraries previously generated by the laboratory of Patrick C. Phillips and obtained from the NCBI SRA in December 2020 (accessions are listed at the end of this article). We eliminated microbial and other contaminants after polishing with the SIDR software (Fierst and Murdock 2017). Briefly, SIDR uses ensemble-based machine learning to train a model of sequence identity (i.e., target or contaminant) based on measured predictor variables. Here, the predictors were sequence GC content, read depth of Nanopore libraries aligned to the assembled sequences and *k*-mer frequency distributions with k=19.

Residual allelism has been a problem with previous *Caenorhabditis* genome sequences (Barriere et al. 2009). We used the purge_haplotigs software version 1.1.1 (Roach et al. 2018) to identify possible heterozygous regions of the assembled sequences. Briefly, we aligned the ONT libraries to the assembled sequences and produced a read-depth histogram to identify regions of abnormal sequencing depth. The method works under the assumption that alleles will result in “split coverage” with read depth approximately 0.5 that of the homozygous contigs. The read-depth histogram did not have noticeable regions of abnormal coverage and the software identified less than 400,000 bp of possible haplotigs in the genome sequences of *C. remanei* PX356, *C. remanei* PX439 and *C. latens* PX534. This represented <0.33% of any of the assembled genome sequences and we chose to retain these sequences in the assembled genome because we could not reliably identify them as “haplotigs.”

In order to estimate error and analyze the potential for false duplications, artifacts and other assembly errors we analyzed weaknesses in our assembled genome sequences. Two of the best indicators of weakness in assembled genome sequences are *k*-mer distributions, short sequences that recur in the assembled sequence and the raw DNA libraries, and sequence read depths (Ko et al. 2022). Unusually high sequence read depths and *k*-mers overrepresented in sequence reads versus the assembled sequence suggest regions of the genome that were incorrectly “collapsed” during assembly whereas unusually low read depths and *k*-mers underrepresented in sequence reads versus the assembled sequence suggest regions of the genome that were falsely duplicated or expanded. We used the software package mosdepth (Pedersen and Quinlan 2018) to analyze sequence read depth across regions of the assembled sequence and the software package KAD (He et al. 2020) to analyze *k*-mer distributions. Both sequence read depth (supplementary figs. S4–S9, Supplementary Material online) and *k*-mer distribution (supplementary figs. S10–S12, table S8, Supplementary Material online) suggested few regions of unusually low sequence or *k*-mer coverage. For example, *k*-mers associated with potentially duplicated or falsely expanded regions were small in number across our three newly assembled sequences (0.0012% of *k*-mers in *C. latens*, 0.002% in *C. remanei* PX439 and 0.0047% in *C. remanei* PX356).

### Creating Pseudo-Molecules

Previous studies have shown remarkable conservation of large-scale synteny between *Caenorhabditis* (Stein et al. 2003; Fierst et al. 2015; Yin et al. 2018; Teterina et al. 2020). We assumed this large-scale synteny is conserved within the interfertile *C. remanei*/*C. latens* species complex and used the chromosome-scale *C. remanei* strain PX506 assembled genome sequence (Teterina et al. 2020) to construct pseudo-molecules for *C. remanei* PX356 and PX439 and *C. latens* PX534 with the RagTag software version 2.1.0 (Alonge et al. 2021). Briefly, RagTag performs homology-based scaffolding by aligning query sequences to a reference assembled sequence with the minimap2 software (Li 2018).

### Gene Annotation

We annotated protein-coding genes in the assembled sequences of *C. remanei* PX356 and PX439 and *C. latens* PX534 with the BRAKER2 v2.1.6 software (Bruna et al. 2021). We used RepeatModeler v2.0.2 (Smit et al. 2013–2015) for *de novo* repeat identification and the queryRepeatDatabase.pl script inside RepeatMasker/util to extract Rhabditida repeats (Bao et al. 2015). We combined these files to create a library of known and *de novo* repeats and used these with RepeatMasker v4.1.2-p1 (Smit et al. 2013–2015) to softmask repeats in the assembled sequence. We aligned RNA-Seq libraries extracted from mixed stage nematode populations to the softmasked sequences with STAR aligner v2.7.9a (Dobin et al. 2013). We used this in BRAKER2 with the protein sequences from *C. remanei* PX506 as homology evidence. Only the RNA-Seq libraries were used for training gene predictors.

We obtained the assembled genome sequences, protein-coding gene annotations and coding sequence files for the remaining *Caenorhabditis* species from WormBase Parasite (Howe et al. 2017) in December 2020 (version WBPS15) with the exception of *C. tropicalis*, which was obtained from the NCBI in February 2021. We performed all of the following analyses (functional annotation, transposable element annotation, whole genome alignment) on the *C. remanei* and *C. latens* assembled genome sequences and protein-coding gene annotations produced by our group and the *Caenorhabditis* assembled genome sequences and protein-coding gene annotations obtained from WormBase Parasite and the NCBI. We used the AGAT suite to calculate gene statistics (Dainat).

Each of the assembled sequences we studied was contained in 6–155 contiguous sequences with the exception of *C. sinica* which is contained in 15,261 sequences. We included *C. sinica* in analyses despite this fragmentation as a representative outcrossing species closer to the root of the Elegans group. We also included the distantly related *Pristionchus pacificus* to orient and root our phylogenetic analyses.

### Functional Annotation

We used the Interproscan software v5.19 (Zdobnov and Apweiler 2001; Finn et al. 2017) to annotate protein domains and motifs, gene ontologies (Consortium 2000) and pathways (Kanehisa and Goto 2000; Kanehisa et al. 2016). Briefly, Interproscan searches multiple databases for protein information including PRINTS (Attwood and Beck 1994; Attwood et al. 1994), Pfam (Punta et al. 2012), ProDom (Bru et al. 2005), and PROSITE (Hulo et al. 2005).

### Whole Genome Alignment

We used ProgressiveCactus version 2.0.4 (Armstrong et al. 2020) to align the *Caenorhabditis* genome sequences and measure the spectrum of mutational events. ProgressiveCactus permits reference-free alignment and uses phylogenetic information to estimate parental (ancestral) genome sequences. The ProgressiveCactus alignment algorithm re-constructs an ancestral sequence for branch points along the phylogeny and mutational events are measured between ancestor and child genomes. ProgressiveCactus defines deletions, insertions, gap deletions and gap insertions by size, where gap deletions and insertions are <5 bp. Transpositions involve transfer of sequence from one chromosomal region to another whereas inversions are regions that have reversed orientation. Duplications are sequences that occurred singly in the ancestor and in multiple copies in the child genome. We used the nematode phylogeny estimated by Ahmed et al. (2022) with *P. pacificus* as an outgroup rooting the alignments.

The output of a ProgressiveCactus (Armstrong et al. 2020) alignment is a binary hierarchical alignment (HAL) file (Hickey et al. 2013). We used the halSummarizeMutations function to calculate substitutions, transitions, transversions, insertions, deletions, duplications and transpositions. The halSummarizeMutations function calculates these quantities for each genome relative to the ancestral genome and for estimated ancestral genomes relative to each estimated parent node. We used the halBranchMutations function to create a BED-formatted file with the genomic locations of each of the mutations and the bedtools intersect function to associate these mutations with annotated repeat elements and protein-coding genes (Quinlan and Hall 2010). We used the hal2fasta function to print the estimated ancestral genome sequences, annotated repeat elements in these with the EDTA software (Ou et al. 2019) and associated mutations in ancestral genomes with annotated repeats using bedtools intersect. We could not reconstruct protein-coding genes in estimated ancestral sequences because the accuracy of protein-coding gene annotation is dependent on nucleotide-level features like start codons and accurate intron-exon boundaries that are not prioritized in Progressive Cactus alignment. However, this approach did allow us to analyze insertions and deletions in repeat elements and specific TE families.

### TE Annotation

We used the Extensive *de novo* TE Annotator software version 2.0 (EDTA; Ou et al. 2019) to identify repeats in *Caenorhabditis* genome sequences. EDTA uses a number of different open-source tools to identify TE candidates in genome sequences and combines these with annotated repeats using known coding sequences (CDS) to eliminate false positive TEs. We used the Rhabditida repeats extracted from RepeatMasker (Bao et al. 2015) and the CDS obtained from BRAKER2 (Bruna et al. 2021) annotation, WormBase Parasite (Howe et al. 2017) and the NCBI. Long terminal repeat retrotransposons (LTRs) were identified with LTR Harvest (Ellinghaus et al. 2008), LTR Finder (Xu and Wang 2007), LTR retriever (Ou and Jiang 2018, 2019) and Generic Repeat Finder (Shi and Liang 2019). Generic Repeat Finder (Shi and Liang 2019) was also used to identify Terminal Direct Repeats (TDRs), Miniature Inverted repeat Transposable Elements (MITEs) and Terminal Inverted Repeats (TIRs). TIRs were also identified with TIR-Learner (Su et al. 2019). Helitron transposons were identified with HelitronScanner (Xiong et al. 2014) and TEsorter used to classify identified TEs (Zhang et al. 2019). Tandem Repeat Finder (TRF) was used to identify tandem repeats (Benson 1999). EDTA (Ou et al. 2019) calculates the number, base pairs and percentage of the genome covered by different TEs but a large proportion of the TIR (DNA) elements were not assigned to families found in the Sequence Ontology database and classified as generic “repeat region.” To accurately characterize these we used the RepeatMasker (Bao et al. 2015) annotation table with the EDTA-identified TEs to calculate the number, base pairs and percentage of the genome covered by different classes and types of TEs.

### Phylogenetic Comparative Analyses of TE Evolution

We followed the phylogenetic comparative analysis outlined above (“Reconstructing ancestral genome size”) and mapped “selfer” and “outcrosser” as discrete states onto the smaller Elegans phylogeny with the R software package Ape (Paradis and Schliep 2018). We used SLOUCH (Hansen et al. 2008; Kopperud et al. 2018) to fit Brownian motion and Ornstein–Uhlenbeck single and separate optima models. For TE content we analyzed differences in total repeat content and number for 21 different classes of repeats including retroelements like SINES, LINES, and LTR elements, DNA transposons like PiggyBac and Mutator, rolling circle/Helitrons, unclassified elements and simple repeats (table 1). The small sample size corrected Akaike Information Criteria (AICc) was used to determine which of the three models best captured TE evolution.

### Orthology Assignment

We used OrthoFinder 2.5.4 (Emms and Kelly 2015) to identify orthologous and paralogous genes in *Caenorhabditis* genomes. We selected the longest isoform for each gene with the OrthoFinder primary_transcript.py. Briefly, OrthoFinder aligns proteomes with DIAMOND (Buchfink et al. 2015, 2021) and uses a Markov Cluster Algorithm to assign proteins to orthogroups or gene families.

OrthoFinder assigned proteins to 24,574 different orthogroups or gene families in the Elegans group. *Caenorhabditis* genome sequences can contain contamination from microbes, fungi and viruses (Fierst and Murdock 2017; Fierst et al. 2017) and we identified multiple orthogroups with phylogenetic patterns suggesting contamination. For example, orthogroups restricted to individual lineages, contained only in species sequenced by the same laboratory or phylogenetically separated but sharing similar sequence characteristics (here, high fragmentation and protein-coding gene counts beyond the Elegans median) could not be conclusively identified as *Caenorhabditis*. Including genes from contaminants would overestimate gene family turnover rates and to avoid this we restricted our analyses to 14,590 high-confidence orthogroups present in at least half the Elegans genomes. Our method allowed us to identify orthogroups lost in self-fertile species as long as these orthogroups were not concurrently lost in multiple outcrossing species.

### Gene Birth and Death Analyses

We calculated gene family expansions and contractions with the CAFE5 software (Mendes et al. 2020). CAFE5 assumes that each gene family has at least one representative at the base of the tree and we eliminated the distantly related *P. pacificus* from the analysis. *C. sinica* has the most fragmented assembled sequence of the species we studied here but its annotated protein-coding gene complement is large enough to fit this requirement and we included it throughout this manuscript to orient our analyses and reduce bias that might be introduced by overly distant species like *P. pacificus*, assembled sequences with retained allelism like the outcrossing *C. brenneri* (Barriere et al. 2009) or possible reduced complements of protein-coding genes like the self-fertile *C. elegans* (Stevens et al. 2019). We used phytools (Revell 2012) to create a dichotomous, ultrametric phylogenetic tree from the Ahmed et al. (2022) estimated phylogeny.

We estimated an error model with the CAFE5 “-e” option (Han et al. 2013) and used this error model in further analyses. CAFE5 uses maximum-likelihood estimation to fit gene birth-death rate parameters or λ values based on a user specifying the number of discrete λ values. Gene families changing significantly are those experiencing rapid expansions or contractions across segments of the phylogenetic tree. We fit models with 2–8 λ values and found that the model with λ=2 had the highest likelihood (here, defined as greater than 2 units following Burnham and Anderson 2002) given the dataset. We used the R statistical software package qvalue (Storey et al. 2019) to correct the CAFE5 significance estimates for multiple comparisons.

To focus on gene families that were expanding or contracting in parallel in selfing and outcrossing lineages we extracted orthogroups that were identified as significant (q<0.01; fdr<0.1) in the CAFE5 analyses and implemented a set of Boolean rules. If an orthogroup was stable or decreasing in all selfing lineages and stable or increasing in all outcrossing lineages we defined it as “Decreasing in Selfers.” Similarly, if an orthogroup was stable or decreasing in all outcrossing lineages and stable or increasing in all selfing lineages we defined it as “Decreasing in Outcrossers.” If an orthogroup was stable or increasing in all selfing lineages and stable or decreasing in all outcrossing lineages we defined it as “Increasing in Selfers.” Similarly, an orthogroup that was stable or decreasing in all outcrossing lineages and stable or increasing in all selfing lineages was “Decreasing in Outcrossers.” We searched the functional annotations for information on any member of these orthogroups and searched *C. elegans* for orthologous genes as these may also provide functional information. We analyzed statistical significance with Fisher’s exact tests comparing the observed magnitude of gene family change with the expected magnitude given the overall gene family size.

## Accession numbers

*C. remanei* PX356 Bioproject PRJNA248909

*C. remanei* PX439 Bioproject PRJNA248911

*C. remanei* PX506 Bioproject PRJNA577507

*C. latens* PX534 Bioproject PRJNA248912

*C. briggsae* AF16 Bioproject PRJNA20855

*C. nigoni* JU1422 Bioproject PRJNA384657

*C. elegans* N2 Bioproject PRJNA158, PRJNA13758

*C. tropicalis* NIC58, JU1373 Bioproject PRJNA662844

*C. inopinata* NKZ35 Bioproject PRJDB5687

*C. sinica* ZZY0401 Bioproject PRJNA194557

## Supplementary Material

msad039_Supplementary_Data

## Data Availability

Bioinformatic scripts, software, workflows, and raw data files associated with this project are located at https://github.com/jannafierst/Worm-nomics and doi:10.17605/OSF.IO/FAJ8Z.

## References

[msad039-B1] Adams PE , Crist AB, Young EM, Wilis JH, Phillips PC, Fierst JL. 2022. Slow recovery from inbreeding depression generated by the complex architecture of segregating deleterious mutations. Mol Biol Evol. 39(1):msab330. 34791426 10.1093/molbev/msab330 PMC8789292

[msad039-B2] Ahmed M , Roberts NG, Adediran F, Smythe AB, Kocot KM, Holovachov O. 2022. Phylogenomic analysis of the Phylum Nematoda: conflicts and congruences with morphology, 18S rRNA, and mitogenomes. Front Ecol Evol. 9:76956.

[msad039-B3] Alonge M , Lebeigle L, Kirsche M, Aganezov S, Wang X, Lippman ZB, Schatz MC, Soyk S. 2021. Automated assembly scaffolding elevates a new tomato system for high-throughput genome editing. *BioRXiv*. 10.1186/s13059-022-02823-7 PMC9753292 36522651

[msad039-B4] Andersen EC , Gerke JP, Shapiro JA, Crissman JR, Ghosh R, Bloom JS, Felix M-A, Kruglyak L. 2012. Chromosome-scale selective sweeps shape *Caenorhabditis elegans* genomic diversity. Nat Genet. 44(3):285–290. 22286215 10.1038/ng.1050 PMC3365839

[msad039-B5] Andersson M . 1994. Sexual selection. Princeton (NJ): Princeton University Press.

[msad039-B6] Armstrong J , Hickey G, Diekhans M, Fiddes IT, Novak AM, Deran A, Fang Q, Xie D, Feng S, Stiller J, et al 2020. Progressive Cactus is a multiple-genome aligner for the thousand-genome era. Nature 587:246–251. 33177663 10.1038/s41586-020-2871-y PMC7673649

[msad039-B7] Attwood TK , Beck ME. 1994. PRINTS: a protein motif fingerprint database. Protein Eng. 7:841–848. 7971946 10.1093/protein/7.7.841

[msad039-B8] Attwood TK , Beck ME, JBleasby A, Parry-Smith DJ. 1994. PRINTS: a database of protein motif fingerprints. Nucleic Acids Res. 22:3590–3596. 7937065 PMC308327

[msad039-B9] Baldi C , Cho S, Ellis RE. 2009. Mutations in two independent pathways are sufficient to create hermaphroditic nematodes. Science 326(5955):1002–1005. 19965511 10.1126/science.1176013

[msad039-B10] Bao W , Kojima KK, Kohany O. 2015. Repbase update, a database of repetitive elements in eukaryotic genomes. Mob DNA. 6:4–9. 26045719 10.1186/s13100-015-0041-9 PMC4455052

[msad039-B11] Barriere A , Yang S-P, Pekarek E, Thomas CG, Haag ES, Ruvinsky I. 2009. Detecting heterozygosity in shotgun genome assemblies: lessons from obligately outcrossing nematodes. Genome Res. 19:470–480. 19204328 10.1101/gr.081851.108 PMC2661809

[msad039-B12] Benson G . 1999. Tandem repeats finder: a program to analyze DNA sequences. Nucleic Acids Res. 27(2):573–580. 9862982 10.1093/nar/27.2.573 PMC148217

[msad039-B13] Bird DM , Blaxter ML, McCarter JP, Mitreva M, Sternberg PW, Thomas WK. 2005. A white paper on nematode comparative genomics. J Nematol. 37(4):408–416. 19262884 PMC2620993

[msad039-B14] Blaxter M , Archibald JM, Childers AK, Coddington JA, Crandall KA, Di Palma F, Durbin R, Edwards SV, Graves JAM, Hackett KJ, et al 2022. Why sequence all eukaryotes? Proc Natl Acad Sci U S A. 119(4):e2115636118. 10.1073/pnas.2115636118 PMC8795522 35042801

[msad039-B15] Blommaert J . 2020. Genome size evolution: towards new model systems for old questions. Proc R Soc B. 287(1933):20201441. 10.1098/rspb.2020.1441 PMC7482279 32842932

[msad039-B16] Boutin TS , Le Rouzic A, Capy P. 2012. How does selfing affect the dynamics of selfish transposable elements? Mob DNA. 3:5. 22394388 10.1186/1759-8753-3-5 PMC3395816

[msad039-B17] Bru C , Courcelle E, Carrere S, Beausse Y, Dalmar S, Kahn D. 2005. The ProDom database of protein domain families: more emphasis on 3D. Nucleic Acids Res. 33(S1):D212–D215. 15608179 10.1093/nar/gki034 PMC539988

[msad039-B18] Bruna T , Hoff KJ, Lomsadze A, Stanke M, Borodovsky M. 2021. BRAKER2: automatic eukaryotic genome annotation with GeneMark-EP+ and AUGUSTUS supported by a protein database. NAR Genom Bioinform. 3(1):lqaa108. 33575650 10.1093/nargab/lqaa108 PMC7787252

[msad039-B19] Buchfink B , Reuter K, Drost HG. 2021. Sensitive protein alignments at tree-of-life scale using DIAMOND. Nat Methods. 18:366–368. 33828273 10.1038/s41592-021-01101-x PMC8026399

[msad039-B20] Buchfink B , Xie C, Huson DH. 2015. Fast and sensitive protein alignment using DIAMOND. Nat Methods. 12:59–60. 25402007 10.1038/nmeth.3176

[msad039-B21] Burnham KP , Anderson DR. 2002. Model selection and multimodel inference: a practical information-theoretic approach. 2nd ed. Berlin, Heidelberg: Springer.

[msad039-B22] Carlton PM , Davis RE, Ahmed S. 2022. Nematode chromosomes. Genetics 221(1):iyac014. 35323874 10.1093/genetics/iyac014 PMC9071541

[msad039-B23] Chandler VL , Walbot V. 1986. DNA modification of a maize transposable element correlates with loss of activity. Proc Natl Acad Sci U S A. 83:1767–1771. 3006070 10.1073/pnas.83.6.1767 PMC323165

[msad039-B24] Chen P , Zhang J. 2021. Asexual experimental evolution of yeast does not curtail transposable elements. Mol Biol Evol. 38(7):2831–2842. 33720342 10.1093/molbev/msab073 PMC8233515

[msad039-B25] Consortium TGO . 2000. Gene ontology: tool for the unification of biology. Nat Genet. 25(1):25–29. 10802651 10.1038/75556 PMC3037419

[msad039-B26] Cook DE , Zdraljevic S, Roberts JP, Andersen EC. 2017. CeNDR, the *Caenorhabditis elegans* natural diversity resource. Nucleic Acids Res. 45:D650–D657. 27701074 10.1093/nar/gkw893 PMC5210618

[msad039-B27] Crombie T , Zdraljevic S, Cook DE, Tanny RE, Brady SC, Wang Y, Evans KS, Hahnel S, Lee D, Rodriguez BC, et al 2019. Deep sampling of Hawaiian *Caenorhabditis elegans* reveals high genetic diversity and admixture with global populations. Elife 8:e50465. 31793880 10.7554/eLife.50465 PMC6927746

[msad039-B28] Cutter AD , Baird SE, Charlesworth D. 2006. High nucleotide polymorphism and rapid decay of linkage disequilibrium in wild populations of *Caenorhabditis remanei*. Genetics 174:901–913. 16951062 10.1534/genetics.106.061879 PMC1602088

[msad039-B29] Cutter AD , Dey A, Murray RL. 2009. Evolution of the *Caenorhabditis elegans* genome. Mol Biol Evol. 26(6):1199–1234. 19289596 10.1093/molbev/msp048

[msad039-B30] Dainat J . ****v0.8.0. AGAT: another GFF analysis toolkit to handle annotations in any GTF/GFF format. *Zenodo*. Available from: 10.5281/zenodo.3552717

[msad039-B31] Denver DR , Morris K, Lynch M, Thomas WK. 2004. High mutation rate and predominance of insertions in the *Caenorhabditis elegans* nuclear genome. Nature 430(7000):679–682. 15295601 10.1038/nature02697

[msad039-B32] Dey A , Chan CKW, Thomas CG, Cutter AD. 2013. Molecular hyperdiversity defines populations of the nematode *Caenorhabditis brenneri*. Proc Natl Acad Sci U S A. 110(27):11056–11060. 23776215 10.1073/pnas.1303057110 PMC3703985

[msad039-B33] Dey A , Jeon Y, Wang G, Cutter AD. 2012. Global population genetic structure of *Caenorhabditis remanei* reveals incipient speciation. Genetics 191:1257–1269. 22649079 10.1534/genetics.112.140418 PMC3416005

[msad039-B34] Dey A , Jin Q, Chen Y, Cutter AD. 2014. Gonad morphogenesis defects drive hybrid male sterility in asymmetric hybrid breakdown of *Caenorhabditis* nematodes. Evol Dev. 16(6):362–372. 25196892 10.1111/ede.12097 PMC4244252

[msad039-B35] Dobin A , Davis CA, Schlesinger F, Drenkow J, Zaleski C, Jha S, Batut P, Chaisson M, Gingeras TR. 2013. STAR: ultrafast universal RNA-seq aligner. Bioinformatics 29(1):15–21. 23104886 10.1093/bioinformatics/bts635 PMC3530905

[msad039-B36] Dolgin ES , Charlesworth B. 2006. The fate of transposable elements in asexual populations. Genetics 174(2):817–827. 16888330 10.1534/genetics.106.060434 PMC1602064

[msad039-B37] Dolgin ES , Charlesworth B, Baird SE, Cutter AD. 2007. Inbreeding and outbreeding depression in *Caenorhabditis* nematodes. Evolution 61(6):1339–1352. 17542844 10.1111/j.1558-5646.2007.00118.x

[msad039-B38] Dupeyron M , Singh KS, Bass C, Hayward A. 2019. Evolution of *Mutator* transposable elements across eukaryotic diversity. Mob DNA. 10:12. 30988700 10.1186/s13100-019-0153-8 PMC6446971

[msad039-B39] Ekblom R , Wolf JBW. 2014. A field guide to whole-genome sequencing, assembly and annotation. Evol Appl. 7:1026–1042. 25553065 10.1111/eva.12178 PMC4231593

[msad039-B40] Ellinghaus D , Kurtz S, Willhoeft U. 2008. LTRharvest, an efficient and flexible software for *de novo* detection of LTR retrotranposons. BMC Bioinformatics 9:18. 18194517 10.1186/1471-2105-9-18 PMC2253517

[msad039-B41] Elliott TA , Gregory TR. 2015. What’s in a genome? The C-value enigma and the evolution of eukaryotic genome content. Philos Trans R Soc B Biol Sci. 370:20140331. 10.1098/rstb.2014.0331 PMC4571570 26323762

[msad039-B42] Emms DM , Kelly S. 2015. OrthoFinder: solving fundamental biases in whole genome comparisons dramatically improves orthogroup inference accuracy. Genome Biol. 16:157. 26243257 10.1186/s13059-015-0721-2 PMC4531804

[msad039-B43] Farslow JC , Lipinski KJ, Packard LB, Edgley ML, Taylor J, Flibotte S, Moerman DG, Katju V, Bergthorsson U. 2015. Rapid increase in frequency of gene copy-number variants during experimental evolution in *Caenorhabditis elegans*. BMC Genomics 16(1):1–18. 26645535 10.1186/s12864-015-2253-2 PMC4673709

[msad039-B44] Felix M-A , Braendle C, Cutter AD. 2014. A streamlined system for species diagnosis in Caenorhabditis (Nematoda: Rhabditidae) with name designations for 15 distinct biological species. PLoS ONE 9(4):e94723. 24727800 10.1371/journal.pone.0094723 PMC3984244

[msad039-B45] Fierst JL , Murdock DA. 2017. Decontaminating eukaryotic genome assemblies with machine learning. BMC Bioinformatics 18(1):533. 29191179 10.1186/s12859-017-1941-0 PMC5709863

[msad039-B46] Fierst JL , Murdock DA, Thanthiriwatte C, Willis JH, Phillips PC. 2017. Metagenome-assembled draft genome sequence of a novel microbial *Stenotrophomonas maltophilia* strain isolated from *Caenorhabditis remanei* tissue. Genome Announc. 5(7):e01646-16. 28209833 10.1128/genomeA.01646-16 PMC5313625

[msad039-B47] Fierst JL , Willis JH, Thomas CG, Wang W, Reynolds RM, Ahearnge TE, Cutter AD, Phillips PC. 2015. Reproductive mode and the evolution of genome size and structure in *Caenorhabditis* nematodes. PLoS Genet. 11(6):e1005323. 26114425 10.1371/journal.pgen.1005323 PMC4482642

[msad039-B48] Finn RD , Attwood TK, Babbitt PC, Bateman A, Bork P, Bridge AJ, Chang HY, Dosztanyi Z, El-Gebali S, Fraser M, et al 2017. Interpro in 2017-beyond protein family and domain annotations. Nucleic Acids Res. 45(D1):D190–D199. 27899635 10.1093/nar/gkw1107 PMC5210578

[msad039-B49] Gregory TR . 2005a. The C-value enigma in plants and animals: a review of parallels and an appeal for partnership. Ann Bot. 95:133–146. 15596463 10.1093/aob/mci009 PMC4246714

[msad039-B50] Gregory TR . 2005b. Genome size evolution in animals. In: Gregory TR, editor. The evolution of the genome. San Diego: Elsevier.

[msad039-B51] Guo Y , Lang S, Ellis RE. 2009. Indepdendent recruitment of F box genes to regulate hermaphrodite development during nematode evolution. Curr Biol. 19:1853–1860. 19836240 10.1016/j.cub.2009.09.042

[msad039-B52] Guo W , Wang D, Lisch D. 2021. RNA-directed DNA methylation prevents rapid and heritable reversal of transposon silencing under heat stress in *Zea mays*. PLoS Genet. 17(6):e1009326. 34125827 10.1371/journal.pgen.1009326 PMC8224964

[msad039-B53] Haag ES , Chamberlin H, Coghlan A, Fitch DH, Peters AD, Schulenburg H. 2007. *Caenorhabditis* evolution: if they all look alike, you aren’t looking hard enough. Trends Genet. 23(3):101–104. 17275130 10.1016/j.tig.2007.01.002

[msad039-B54] Hahn MW , De Bie T, Stajich JE, Nguyen C, Cristianini N. 2005. Estimating the tempo and mode of gene family evolution from comparative genomic data. Genome Res. 15:1153–1160. 16077014 10.1101/gr.3567505 PMC1182228

[msad039-B55] Hahn MW , Han MV, Han S. 2007. Gene family evolution across 12 *Drosophila* genomes. PLoS Genet. 3(11):e197. 17997610 10.1371/journal.pgen.0030197 PMC2065885

[msad039-B56] Han MV , Thomas GWC, Lugo-Martinez J, Hahn MW. 2013. Estimating gene gain and loss rates in the presence of error in genome assembly and annotation using CAFE 3. Mol Biol Evol. 30:1987–1997. 23709260 10.1093/molbev/mst100

[msad039-B57] Hansen TF . 1997. Stabilizing selection and the comparative analysis of adaptation. Evolution 51(5):1341–1351. 28568616 10.1111/j.1558-5646.1997.tb01457.x

[msad039-B58] Hansen TF . 2014. Use and misuse of comparative methods in the study of adaptation. In: Garamszegi LZ, editor. Modern phylogenetic comparative methods and their application in evolutionary biology. Berlin, Heidelberg: Springer.

[msad039-B59] Hansen TF , Pienaar J, Orzack SH. 2008. A comparative method for studying adaptation to a randomly evolving environment. Evolution 62(8):1965–1977. 18452574 10.1111/j.1558-5646.2008.00412.x

[msad039-B60] He C , Lin G, Wei H, Tang H, White FF, Valent B, Liu S. 2020. Factorial estimating assembly base errors using *k*-mer abundance difference (KAD) between short reads and genome assembled sequences. NAR Genom Bioinform. 2(3):lqaa075. 33575622 10.1093/nargab/lqaa075 PMC7671381

[msad039-B61] He Y , Tian S, Tian P. 2019. Fundamental asymmetry of insertions and deletions in genomes size evolution. J Theor Biol. 482:109983. 31445016 10.1016/j.jtbi.2019.08.014

[msad039-B62] Hickey G , Paten B, Earl D, Zerbino D, Haussler D. 2013. HAL: a hierarchical format for storing and analyzing multiple genome alignments. Bioinformatics 29(10):1341–1342. 23505295 10.1093/bioinformatics/btt128 PMC3654707

[msad039-B63] Howe KL , Bolt BJ, Shafie M, Kersey P, Berriman M. 2017. WormBase ParaSite: a comprehensive resource for helminth genomics. Mol Biochem Parasitol. 215:2–10. 27899279 10.1016/j.molbiopara.2016.11.005 PMC5486357

[msad039-B64] Hu TT , Pattyn P, Bakker EG, Cao J, Cheng J-F, Clark RM, Fahlgren N, Fawcett JA, Grimwood J, Gundlach H, et al 2011. The *Arabidopsis lyrata* genome sequence and the basis of rapid genome size change. Nat Genet. 43(5):476–481. 21478890 10.1038/ng.807 PMC3083492

[msad039-B65] Hulo N , Bairoch A, Bulliard V, Cerutti L, De Castro E, Lagendijk-Genevaux PS. 2005. The PROSITE database. Nucleic Acids Res. 34(s1):D227–D230. 10.1093/nar/gkj063 PMC1347426 16381852

[msad039-B66] Jiang N , Ferguson AA, Slotkin RK, Lisch D. 2011. Pack-mutator-like transposable elements (pack-MULEs) induce directional modification of genes through biased insertion and DNA acquisition. Proc Natl Acad Sci U S A. 108(4):1537–1542. 21220310 10.1073/pnas.1010814108 PMC3029777

[msad039-B67] Kanehisa M , Goto S. 2000. KEGG: Kyoto encyclopedia of genes and genomes. Nucleic Acids Res. 28(1):27–30. 10592173 10.1093/nar/28.1.27 PMC102409

[msad039-B68] Kanehisa M , Sato Y, Kawashima M, Furumichi M, Tanabe M. 2016. KEGG as a reference resource for gene and protein annotation. Nucleic Acids Res. 44(D1):D457–D462. 26476454 10.1093/nar/gkv1070 PMC4702792

[msad039-B69] Kanzaki N , Tsai IJ, Tanaka R, Hunt VL, Liu D, Tsuyama K, Maeda Y, Namai S, Kumagai R, Tracey A, et al 2018. Biology and genome of a newly discovered sibling species of *Caenorhabditis elegans*. Nat Commun. 9:3216. 30097582 10.1038/s41467-018-05712-5 PMC6086898

[msad039-B70] Kapusta A , Suh A, Feschotte C. 2017. Dynamics of genome size evolution in birds and mammals. Proc Natl Acad Sci U S A. 114(8):E1460–E1469. 28179571 10.1073/pnas.1616702114 PMC5338432

[msad039-B71] Katju V , Bergthorsson U. 2013. Copy-number changes in evolution: rates, fitness effects and adaptive significance. Front Genet. 4:273. 24368910 10.3389/fgene.2013.00273 PMC3857721

[msad039-B72] Kim C , Kim J, Kim S, Cook DE, Evans KS, Andersen EC, Lee J. 2019. Long-read sequencing reveals intra-species tolerance of substantial structural variations and new subtelomere formation in *C. elegans*. Genome Res. 29:1023–1035. 31123081 10.1101/gr.246082.118 PMC6581047

[msad039-B73] Kiontke KC , Felix MA, Ailion M, Rockman MV, Braendle C, Penigault JB, Fitch DHA. 2011. A phylogeny and molecular barcodes for *Caenorhabditis*, with numerous new species from rotting fruits. BMC Evol Biol. 11:339. 22103856 10.1186/1471-2148-11-339 PMC3277298

[msad039-B74] Kiontke K , Fitch DH. 2005. The phylogenetic relationships of *Caenorhabditis* and other rhabditids. In: WormBook. 11th ed. The *C. elegans* Research Community. p. 1–11. 10.1895/wormbook.1.11.1 PMC4781183 18050394

[msad039-B75] Kiontke K , Gavin NP, Raynes Y, Roehrig C, Piano F, Fitch DHA. 2004. *Caenorhabditis* phylogeny predicts convergence of hermaphroditism and extensive intron loss. Proc Natl Acad Sci U S A. 101(24):9003–9008. 15184656 10.1073/pnas.0403094101 PMC428462

[msad039-B76] Kipreos ET , Pagano M. 2000. The F-box protein family. Genome Biol. 1(5):REVIEWS3002. 10.1186/gb-2000-1-5-reviews3002 PMC138887 11178263

[msad039-B77] Ko BJ , Lee C, Kim J, Rhie A, Yoo DA, Howe K, Wood J, Cho S, Brown S, Formenti G, et al 2022. Widespread false gene gains caused by duplication errors in genome assemblies. Genome Biol. 23:205. 36167596 10.1186/s13059-022-02764-1 PMC9516828

[msad039-B78] Kolmogorov M , Yuan J, Lin Y, Pevzner PA. 2019. Assembly of long, error-prone reads using repeat graphs. Nat Biotechnol. 37:540–546. 30936562 10.1038/s41587-019-0072-8

[msad039-B79] Konrad A , Flibotte S, Taylor J, Waterston RH, Moerman DG, Bergthorsson U, Katju V. 2018. Mutational and trancriptional landscape of spontaneous gene duplications and deletions in Caenorhabditis elegans. Proc Natl Acad Sci U S A. 115(28):7386–7391. 29941601 10.1073/pnas.1801930115 PMC6048555

[msad039-B80] Kopperud BT , Pienaar J, Voje KL, Orzack SH, Hansen TF, Grabowski M. 2018. SLOUCH v2.0: stochastic linear Ornstein–Uhlenbeck models for comparative hypotheses. Available from: https://cran.r-project.org/web/packages/slouch/index.html

[msad039-B81] Koren S , Walenz BP, Berlin K, Miller JR, Bergman NH, Phillippy AM. 2017. Canu: scalable and accurate long-read assembly via adaptive *k*-ner weighting and repeat separation. Genome Res. 27(5):722–736. 28298431 10.1101/gr.215087.116 PMC5411767

[msad039-B82] Lee BY , Kim J, Lee J. 2022. Intraspecific de novo gene birth revealed by presence–absence variant genes in *Caenorhabditis elegans*. NAR Genom Bioinform. 4(2):lqac031. 35464238 10.1093/nargab/lqac031 PMC9022459

[msad039-B83] Lee D , Zdraljevic S, Stevens L, Wang Y, Tanny RE, Crombie TA, Cook DE, Webster AK, Chirakar R, Baugh LR, et al 2021. Balancing selection maintains hyper-divergent haplotypes in *Caenorhabditis elegans*. Nat Ecol Evol. 5:794–807. 33820969 10.1038/s41559-021-01435-x PMC8202730

[msad039-B84] Li H . 2018. Minimap2: pairwise alignment for nucleotide sequences. Bioinformatics 34(18):3094–3100. 29750242 10.1093/bioinformatics/bty191 PMC6137996

[msad039-B85] Lipinski KJ , Farslow JC, Fitzpatrick KA, Lynch M, Katju V, Bergthorsson U. 2011. High spontaneous rate of gene duplication in *Caenorhabditis elegans*. Curr Biol. 21(4):306–310. 21295484 10.1016/j.cub.2011.01.026 PMC3056611

[msad039-B86] Lisch D . 2015. Mutator and MULE transposons. Microbiol Spectr. 3(2):MDNA3-0032. 10.1128/microbiolspec.MDNA3-0032-2014 26104710

[msad039-B87] Liu K , Wessler SR. 2017. Functional characterization of the active mutator-like transposable element, muta1 from the mosquito *Aedes aegypti*. Mob DNA. 8:1–12. 28096902 10.1186/s13100-016-0084-6 PMC5225508

[msad039-B88] Lynch M , Conery JS. 2003. The origins of genome complexity. Science 302:1401–1404. 14631042 10.1126/science.1089370

[msad039-B89] Ma F , Lau CY, Zheng C. 2021. Large genetic diversity and strong positive selection in F-box and GPCR genes among the wild isolates of *Caenorhabditis elegans*. Genome Biol Evol. 13(5), evab048. 33693740 10.1093/gbe/evab048 PMC8120010

[msad039-B90] Mahler DL , Ingram T. 2014. Phylogenetic comparative methods for studying clade-wide convergence. In: Garamszegi LZ, editor. Modern phylogenetic comparative methods and their application in evolutionary biology. Berlin, Heidelberg: Springer. p. 425–450.

[msad039-B91] Makalowski W , Gotea V, Pande A, Makalowska I. 2019. Transposable elements: classification, identification, and their use as a tool for comparative genomics. Methods Mol Biol. 1910:177–207. 31278665 10.1007/978-1-4939-9074-0_6

[msad039-B92] Martienssen R , Baron A. 1994. Coordinate suppression of mutations caused by robertson’s mutator transposons in maize. Genetics 136:1157–1170. 8005422 10.1093/genetics/136.3.1157 PMC1205871

[msad039-B93] Maydan JS , Lorch A, LEdgley M, Flibotte S, Moerman DG. 2010. Copy number variation in the genomes of twelve natural isolates of *Caenorhabditis elegans*. BMC Genomics 11:62. 20100350 10.1186/1471-2164-11-62 PMC2822765

[msad039-B94] Mefford HC . 2014. Shedding light on the genome’s dark matter. Sci Transl Med. 6(257):257.

[msad039-B95] Mendes FK , Vanderpool D, Fulton B, Hahn MW. 2020. CAFE 5 models variation in evolutionary rates among gene families. Bioinformatics 36:5516–5518. 10.1093/bioinformatics/btaa1022 33325502

[msad039-B96] Noble LM , Yuen J, Stevens L, Moya N, Persaud R, Moscatelli M, Jackson JL, Zhang G, Chitrakar R, Baugh LR, et al 2021. Selfing is the safest sex for *caenorhabdtis tropicalis*. Elife 10:e62587. 33427200 10.7554/eLife.62587 PMC7853720

[msad039-B97] Nowell RM , Wilson CG, Almeida P, Schiffer PH, Fontaneto D, Becks L, Rodriguez F, Arkhipova IR, Barraclough TG. 2021. Evolutionary dynamics of transposable elements in bdelloid rotifers. Elife 10:e63194. 33543711 10.7554/eLife.63194 PMC7943196

[msad039-B98] O’Meara BC , Beaulieu J. 2014. Modelling stabilizing selection: the attraction of Orstein–Uhlenbeck models. In: Garamszegi LZ, editor. Modern phylogenetic comparative methods and their application in evolutionary biology. Berlin, Heidelberg: Springer. p. 381–393.

[msad039-B99] Ou S , Jiang N. 2018. LTR retriever: a highly accurate and sensitive program for identification of long terminal repeat retrotransposons. Plant Physiol. 180(4):1803–1815. 10.1104/pp.17.01310 PMC5813529 29233850

[msad039-B100] Ou S , Jiang N. 2019. LTR FINDER parallel: parallelization of LTR FINDER enabling rapid identification of long terminal repeat retrotranposons. Mob DNA. 10(1):48. 31857828 10.1186/s13100-019-0193-0 PMC6909508

[msad039-B101] Ou S , Su W, Liao Y, CHougule K, Agda JRA, Hellinga AJ, Santiago C, Lugo B, Elliott TA, Ware D, et al 2019. Benchmarking transposable element annotation methods for creation of a streamlined, comprehensive pipeline. Genome Biol. 20(1):275. 31843001 10.1186/s13059-019-1905-y PMC6913007

[msad039-B102] Paradis E , Schliep K. 2018. ape 5.0: an environment for modern phylogenetics and evolutionary analyses in R. Bioinformatics 35(3):526–528. 10.1093/bioinformatics/bty633 30016406

[msad039-B103] Pedersen BS , Quinlan AR. 2018. Mosdepth: quick coverage calculation for genomes and exomes. Bioinformatics 34(5):867–868. 29096012 10.1093/bioinformatics/btx699 PMC6030888

[msad039-B104] Pflug JM , Holmes VR, Burrus C, Johnston JS, Maddison DR. 2020. Measuring genome sizes using read-depth, k-mers, and flow cytometry: methodological comparisons in beetles (coleoptera). G3 10(9):3047–3060. 32601059 10.1534/g3.120.401028 PMC7466995

[msad039-B105] Prabh N , Roeseler W, Witte H, Eberhardt G, Sommer RJ, Rodelsperger C. 2018. Deep taxon sampling reveals the evolutionary dynamics of novel gene families in *Pristionchus* nematodes. Genome Res. 28:1664–1674. 30232197 10.1101/gr.234971.118 PMC6211646

[msad039-B106] Punta M , Coggill PC, Eberhardt RY, Mistry J, Tate J, Boursnell C, Pang N, Forslund K, Ceric G, Clements J, et al 2012. The Pfam protein families database. Nucleic Acids Res. 40(D1):D290–D301. 22127870 10.1093/nar/gkr1065 PMC3245129

[msad039-B107] Quinlan AR , Hall IM. 2010. BEDTools: a flexible suite of utilities for comparing genomic features. Bioinformatics 26(6):841–842. 20110278 10.1093/bioinformatics/btq033 PMC2832824

[msad039-B108] Revell LJ . 2012. phytools: an R package for phylogenetic comparative biology (and other things). Methods Ecol Evol. 3(2):217–223.

[msad039-B109] Roach MJ , Schmidt SA, Borneman AR. 2018. Purge halotigs: allelic contig reassignment for third-gen diploid genome assemblies. BMC Bioinformatics 19:460. 30497373 10.1186/s12859-018-2485-7 PMC6267036

[msad039-B110] Robertson DS . 1978. Characterization of a mutator system in maize. Mutat Res. 51:21–28.

[msad039-B111] Robertson DS . 1986. Genetic studies on the loss of mu mutator activity in maize. Genetics 113:765–773. 17246337 10.1093/genetics/113.3.765 PMC1202869

[msad039-B112] Rodelsperger C , Roseler W, Prabh N, Yoshida K, Weiler C, Herrmann M, Sommer RJ. 2018. Phylotranscriptomics of *Pristionchus* nematodes reveals parallel gene loss in six hermaphroditic lineages. Curr Biol. 28(19):3123–3127.e5. 30245109 10.1016/j.cub.2018.07.041

[msad039-B113] Roessler K , Muyle A, Diez CM, Gaut GRJ, Bousios A, Stitzer MC, Seymour DK, Doebley JF, Liu Q, Gaut BS. 2019. The genome-wide dynamics of purging during selfing in maize. Nat Plants. 5(9):980–990. 31477888 10.1038/s41477-019-0508-7

[msad039-B114] Sambrook J . 2001. Molecular cloning: a laboratory manual. 3rd ed. Cold Spring Harbor (NY): Cold Spring Harbor Laboratory Press.

[msad039-B115] Schrader L , Pan H, Bollazzi M, Schiott M, Larabee FJ, Bi X, Deng Y, Zhang G, Boomsma JJ, Rabeling C. 2021. Relaxed selection underlies genome erosion in socially parasitic ant species. Nat Commun. 12:2918. 34006882 10.1038/s41467-021-23178-w PMC8131649

[msad039-B116] Shi J , Liang C. 2019. Generic repeat finder: a high-sensitivity tool for genome-wide de novo repeat detection. Plant Physiol. 180(4):1803–1815. 31152127 10.1104/pp.19.00386 PMC6670090

[msad039-B117] Shimizu KK , Tsuchimatsu T. 2015. Evolution of selfing: recurrent patterns in molecular adaptation. Annu Rev Ecol Evol Syst. 46:593–622.

[msad039-B118] Shuster SM , Wade J. 2003. Mating systems and strategies. Princeton (NJ): Princeton University Press.

[msad039-B119] Simpson VJ , Johnson TE, Hammen RF. 1986. *Caenorhabditis elegans* DNA does not contain 5-methylcytosine at any time during development or aging. Nucleic Acids Res. 14:6711–6719. 3748820 10.1093/nar/14.16.6711 PMC311675

[msad039-B120] Slotkin RK . 2005. The heritable epigenetic silence of mutator transposons by Mu killer [PhD thesis]. [Berkeley (CA)]: University of California at Berkeley.

[msad039-B121] Slotte T , Hazzouri KM, Ågren JA, Koenig D, Maumus F, Guo Y-L, Steige K, Platts AE, Escobar JS, Newman LK, et al 2013. The *Capsella rubella* genome and the genomic consequences of rapid mating system evolution. Nat Genet. 45(7):831–835. 23749190 10.1038/ng.2669

[msad039-B122] Smit AFA , Hubley R, Green P. 2013–2015. Repeatmasker open-4.0. Available from: http://www.repeatmasker.org

[msad039-B123] Stamatakis A . 2014. RAxML version 8: a tool for phylogenetic analysis and post-analysis of large phylogenies. Bioinformatics 30(9):1312–1313. 24451623 10.1093/bioinformatics/btu033 PMC3998144

[msad039-B124] Stein LD , Bao Z, Blasiar D, Blumenthal T, Brent MR, Chen N, Chinwalla A, Clarke L, Clee C, Coghlan A, et al 2003. The genome sequence of *Caenorhabditis briggsae*: a platform for comparative genomics. PLoS Biol. 1(2):E45. 14624247 10.1371/journal.pbio.0000045 PMC261899

[msad039-B125] Stevens L , Felix M, Beltran T, Braendle C, Caurcel C, Fausett S, Fitch D, Frezal L, Gosse C, Kaur T, et al 2019. Comparative genomics of 10 new *Caenorhabditis* species. Evol Lett. 3(2):217–236. 31007946 10.1002/evl3.110 PMC6457397

[msad039-B126] Stiernagle T . 2006. Maintenance of C. elegans. WormBook. 1–11. doi:10.1895/wormbook.1.101.1 PMC4781397 18050451

[msad039-B127] Storey J , Bass A, Dabney A, Robinson D. 2019. qvalue: Q-value estimation for false discovery rate control. R package version 2.14.1. Available from: http://github.com/jdstorey/qvalue

[msad039-B128] Su W , Gu X, Peterson T. 2019. TIR-Learner, a new ensemble method for TIR transposable element annotation, provides evidence for abundant new transposable elements in the maize genome. Mol Plant. 12:447–460. 30802553 10.1016/j.molp.2019.02.008

[msad039-B129] Sutton JM , Millwood JD, MCCormack AC, Fierst JL. 2021. Optimizing experimental design for genome sequencing and assembly with Oxford nanopore technologies. *GigaByte*. doi:10.46471/gigabyte.27 PMC9650304 36824342

[msad039-B130] Teterina A , Willis J, Phillips P. 2020. Chromosome-level assembly of the *Caenorhabditis remanei* genome reveals conserved patterns of nematode genome organization. Genetics 214(4):769–780. 32111628 10.1534/genetics.119.303018 PMC7153949

[msad039-B131] Thomas CG , Li R, Smith HE, Woodruff GC, Oliver B, Haag ES. 2012. Simplification and desexualization of gene expression in self-fertile nematodes. Curr Biol. 22(22):2167–2172. 23103191 10.1016/j.cub.2012.09.038 PMC3568512

[msad039-B132] Thompson OA , Snoek LB, Nijveen H, Sterken MG, Volkers RJM, Brenchley R, Bevers RPJ, Cossins AR, Yanai I, Hajnal A, et al 2015. Remarkably divergent regions punctuate the genome assembly of the *Caenorhabditis elegans* hawaiian strain CB4856. Genetics 200(3):975–989. 25995208 10.1534/genetics.115.175950 PMC4512556

[msad039-B133] Walker BJ , Abeel T, Shea T, Priest M, Abouelliel A, Sakthikumar S, Cuomo CA, Zeng Q, Wortman J, Young SK, et al 2014. Pilon: an integrated tool for comprehensive microbial variant detection and genome assembly improvement. PLoS ONE. 9(11):e112963. 25409509 10.1371/journal.pone.0112963 PMC4237348

[msad039-B134] Wang A , Chen W, Tao S. 2021. Genome-wide characterization, evolution, structure, and expression analysis of the F-box genes in *Caenorhabditis*. BMC Genomics 22:889. 34895149 10.1186/s12864-021-08189-7 PMC8665587

[msad039-B135] Wenzel D , Palladino F, Jedrusik-Bode M. 2011. Epigenetics in *C. elegans*: facts and challenges. Genetics 49:647–661. 10.1002/dvg.20762 21538806

[msad039-B136] Xiong W , He L, Lai J, Dooner HK, Du C. 2014. HelitronScanner uncovers a large overlooked cache of Helitron transposons in many plant genomes. Proc Natl Acad Sci U S A. 111:10263–10268. 24982153 10.1073/pnas.1410068111 PMC4104883

[msad039-B137] Xu Z , Wang H. 2007. LTR FINDER: an efficient tool for the prediction of full-length LTR retrotranposons. Nucleic Acids Res. 35:W265-8. 17485477 10.1093/nar/gkm286 PMC1933203

[msad039-B138] Yin D , Haag ES. 2019. Evolution of sex ratio through gene loss. Proc Natl Acad Sci U S A. 116(26):12919–12924. 31189601 10.1073/pnas.1903925116 PMC6601293

[msad039-B139] Yin D , Schwarz EM, Thomas CG, Felde RL, Korf IF, Cutter AD, Schartner CM, Ralston EJ, Meyer BJ, Haag ES. 2018. Rapid genome shrinkage in a self-fertile nematode reveals sperm competition proteins. Science 359(6371):55–61. 29302007 10.1126/science.aao0827 PMC5789457

[msad039-B140] Zdobnov EM , Apweiler R. 2001. InterProScan: an integration platform for the signature-recognition methods in InterPro. Bioinformatics 17:847–848. 11590104 10.1093/bioinformatics/17.9.847

[msad039-B141] Zhang R , Wang Z, Ou S, Li G. 2019. TEsorter: lineage-level classification of transposable elements using conserved protein domains. *BioRXiv*.

