# Supplementary material for: Genome Size Changes by Duplication, Divergence, and Insertion in Caenorhabditis Worms
Source: Mol Biol Evol. 2023 Feb 20;40(3):msad039. doi: 10.1093/molbev/msad039 (PMC10015627; doi:10.1093/molbev/msad039)
Supplement: msad039_Supplementary_Data [file msad039_supplementary_data.pdf]

Journal Title Here, XXXX, 1–4
doi: DOI HERE
Advance Access Publication Date: Day Month Year
Paper

Genome size changes by duplication, insertion and divergence in Caenorhabditis worms

Paula E. Adams,[1,2] Victoria K. Eggers,[3] Joshua D. Millwood,[1] John M. Sutton,[1,4] Jason Pienaar[3,5] and Janna L. Fierst[3,6]*

[1]Department of Biological Sciences, The University of Alabama, Tuscaloosa, AL 35487, [2]Current address: Department of Biological Sciences, Auburn University, Auburn, AL 36830, [3]Current address: Absci, Vancouver, WA 98663, [4]Institute of the Environment, [5]Biomolecular Sciences Institute and [6]Department of Biological Sciences, Florida International University, Miami, FL 33199

*Corresponding author. jfierst@fiu.edu

FOR PUBLISHER ONLY Received on Date Month Year; revised on Date Month Year; accepted on Date Month Year

Abstract

© The Author XXXX. Published by Oxford University Press. All rights reserved. For permissions, please e-mail: journals.permissions@oup.com

Table 1. Estimated ancestral genome sizes for nodes within the phylogeny with lower and upper 95% Confidence Intervals.

Node	Reconstructed Genome Size	95 % CI lower bound	95 % CI upper bound
49	154.05	127.19	180.92
50	114.51	97.96	131.05
51	110.01	94.31	125.71
52	102.66	89.71	115.60
53	99.05	87.70	110.40
54	91.58	81.87	101.30
55	98.49	87.30	109.68
56	98.17	86.71	109.62
57	94.05	85.42	102.68
58	106.89	99.88	113.90
59	111.53	105.24	117.82
60	114.66	108.47	120.84
61	115.56	109.52	121.60
62	114.11	108.92	119.31
63	110.30	105.25	115.35
64	120.52	117.40	123.63
65	126.79	124.82	128.76
66	123.70	120.56	126.84
67	127.41	125.41	129.41
68	127.60	125.73	129.48
69	111.83	105.10	118.56
70	107.34	99.89	114.80
71	86.92	78.52	95.33
72	82.02	74.89	89.15
73	80.75	76.28	85.23
74	70.33	63.51	77.15
75	195.16	178.84	211.49
76	204.70	191.00	218.39
77	190.08	188.42	191.74
78	228.68	218.05	239.31
79	234.16	225.80	242.51
80	252.73	250.83	254.63
81	232.96	224.90	241.02
82	209.21	203.29	215.13
83	203.71	198.26	209.15
84	201.40	196.29	206.52
85	242.15	240.42	243.87
86	190.45	186.49	194.41
87	186.44	182.82	190.07
88	171.46	168.12	174.79
89	172.83	171.14	174.52
90	172.89	170.37	175.41
91	147.76	146.75	148.76
92	196.52	193.96	199.07
93	210.92	208.72	213.11
94	281.11	278.80	283.42
95	239.81	237.67	241.96

Table 2. Assembly statistics for the *Caenorhabditis* species and strains. Assembled sequence and contig # are raw assembly output and pseudomolecule contig # are after homology-based scaffolding using the *C. remanei* PX506 sequence as a reference. The *C. remanei* PX506 pseudomolecules were generated with Hi-C technology.

	Assembled sequence (Mb)	Contig #	Pseudomolecule sequence (Mb)	Scaffold #
C. remanei PX506	130.48	197	124.80	6
C. remanei PX356	124.50	70	124.07	17
C. remanei PX439	132.05	53	132.00	17
C. latens	120.37	97	119.66	38
C. briggsae	105.42	12		
C. elegans	100.81	7		
C. tropicalis NIC58	81.32	7		
C. tropicalis JU1373	80.98	44		
C. inopinata	122.58	6		
C. nigoni	129.44	155		
C. sinica	130.39	15,261		

Table 3. Genome size, number of protein-coding genes, repeat content and gene content for *Caenorhabditis* genomes

Species	Statistic	Value
C. remanei PX506	Genome Size (Mb)	130.48
C. remanei PX439	Genome Size (Mb)	132.05
C. remanei PX356	Genome Size (Mb)	124.5
C. latens	Genome Size (Mb)	120.37
C. briggsae	Genome Size (Mb)	105.42
C. elegans	Genome Size (Mb)	100.81
C. tropicalis NIC58	Genome Size (Mb)	81.32
C. tropicalis JU1373	Genome Size (Mb)	80.98
C. inopinata	Genome Size (Mb)	122.58
C. nigoni	Genome Size (Mb)	129.44
C. sinica	Genome Size (Mb)	130.39
C. remanei PX506	Protein-coding genes (#)	26189
C. remanei PX439	Protein-coding genes (#)	25045
C. remanei PX356	Protein-coding genes (#)	25977
C. latens	Protein-coding genes (#)	23729
C. briggsae	Protein-coding genes (#)	20821
C. elegans	Protein-coding genes (#)	19997
C. tropicalis NIC58	Protein-coding genes (#)	21210
C. tropicalis JU1373	Protein-coding genes (#)	20829
C. inopinata	Protein-coding genes (#)	21443
C. nigoni	Protein-coding genes (#)	29167
C. sinica	Protein-coding genes (#)	34696
C. remanei PX506	Repeat content (%)	20.23
C. remanei PX439	Repeat content (%)	16.62
C. remanei PX356	Repeat content (%)	16.7
C. latens	Repeat content (%)	13.39
C. briggsae	Repeat content (%)	21.78
C. elegans	Repeat content (%)	14.86
C. tropicalis NIC58	Repeat content (%)	9.79
C. tropicalis JU1373	Repeat content (%)	9.7
C. inopinata	Repeat content (%)	27.1
C. nigoni	Repeat content (%)	27.54
C. sinica	Repeat content (%)	10.41
C. remanei PX506	Gene content (%)	38
C. remanei PX439	Gene content (%)	37.96
C. remanei PX356	Gene content (%)	37.96
C. latens	Gene content (%)	38.53
C. briggsae	Gene content (%)	37.35
C. elegans	Gene content (%)	35.44
C. tropicalis NIC58	Gene content (%)	37.73
C. tropicalis JU1373	Gene content (%)	37.8
C. inopinata	Gene content (%)	38.47
C. nigoni	Gene content (%)	37.75
C. sinica	Gene content (%)	39.48

Table 4. Gene, intergenic, exon and intron content for *Caenorhabditis* genomes

Species	Statistic	Value
C. remanei PX506	Gene content (Mb)	56.396024
C. remanei PX439	Gene content (Mb)	68.123178
C. remanei PX356	Gene content (Mb)	66.196043
C. latens	Gene content (Mb)	64.391427
C. briggsae	Gene content (Mb)	63.979001
C. elegans	Gene content (Mb)	61.897246
C. tropicalis NIC58	Gene content (Mb)	48.863134
C. tropicalis JU1373	Gene content (Mb)	48.959594
C. inopinata	Gene content (Mb)	65.611404
C. nigoni	Gene content (Mb)	63.610343
C. sinica	Gene content (Mb)	67475768
C. remanei PX506	Intergenic content (Mb)	36.54803188
C. remanei PX439	Intergenic content (Mb)	31.79107308
C. remanei PX356	Intergenic content (Mb)	30.13048755
C. latens	Intergenic content (Mb)	35.97948492
C. briggsae	Intergenic content (Mb)	16.67599602
C. elegans	Intergenic content (Mb)	21.21993552
C. tropicalis NIC58	Intergenic content (Mb)	29.86903099
C. tropicalis JU1373	Intergenic content (Mb)	29.62824154
C. inopinata	Intergenic content (Mb)	19.37462555
C. nigoni	Intergenic content (Mb)	23.31727519
C. sinica	Intergenic content (Mb)	37.65278242
C. remanei PX506	Total exon length (Mb)	29.428456
C. remanei PX439	Total exon length (Mb)	32.018437
C. remanei PX356	Total exon length (Mb)	32.319456
C. latens	Total exon length (Mb)	31.137297
C. briggsae	Total exon length (Mb)	29.443465
C. elegans	Total exon length (Mb)	28.708707
C. tropicalis NIC58	Total exon length (Mb)	27.594024
C. tropicalis JU1373	Total exon length (Mb)	27.315355
C. inopinata	Total exon length (Mb)	26.447743
C. nigoni	Total exon length (Mb)	33.294327
C. sinica	Total exon length (Mb)	39.405857
C. remanei PX506	Total intron length (Mb)	26.967568
C. remanei PX439	Total intron length (Mb)	33.878738
C. remanei PX356	Total intron length (Mb)	31.501567
C. latens	Total intron length (Mb)	31.443486
C. briggsae	Total intron length (Mb)	31.896593
C. elegans	Total intron length (Mb)	31.989422
C. tropicalis NIC58	Total intron length (Mb)	21.269825
C. tropicalis JU1373	Total intron length (Mb)	21.644239
C. inopinata	Total intron length (Mb)	39.163661
C. nigoni	Total intron length (Mb)	29.836546
C. sinica	Total intron length (Mb)	28.069911

Table 5. Summed insertions by genomic location for *Caenorhabditis* genomes

Species	Statistic	Value
C. remanei PX506	Insertions in genes (bp)	1990490
C. remanei PX439	Insertions in genes (bp)	2574079
C. remanei PX356	Insertions in genes (bp)	2441169
C. latens	Insertions in genes (bp)	5096024
C. briggsae	Insertions in genes (bp)	7916695
C. elegans	Insertions in genes (bp)	27628203
C. tropicalis NIC58	Insertions in genes (bp)	519494
C. tropicalis JU1373	Insertions in genes (bp)	312084
C. inopinata	Insertions in genes (bp)	33478705
C. nigoni	Insertions in genes (bp)	10572597
C. sinica	Insertions in genes (bp)	36781937
C. remanei PX506	Insertions in TEs (bp)	5144032
C. remanei PX439	Insertions in TEs (bp)	2318260
C. remanei PX356	Insertions in TEs (bp)	2203882
C. latens	Insertions in TEs (bp)	259290
C. briggsae	Insertions in TEs (bp)	302741
C. elegans	Insertions in TEs (bp)	112079
C. tropicalis NIC58	Insertions in TEs (bp)	653800
C. tropicalis JU1373	Insertions in TEs (bp)	298965
C. inopinata	Insertions in TEs (bp)	131448
C. nigoni	Insertions in TEs (bp)	219103
C. sinica	Insertions in TEs (bp)	2991044
C. remanei PX506	Insertions in unannotated regions (bp)	29999
C. remanei PX439	Insertions in unannotated regions (bp)	3337553
C. remanei PX356	Insertions in unannotated regions (bp)	2268088
C. latens	Insertions in unannotated regions (bp)	9051369
C. briggsae	Insertions in unannotated regions (bp)	9064782
C. elegans	Insertions in unannotated regions (bp)	15877634
C. tropicalis NIC58	Insertions in unannotated regions (bp)	4294
C. tropicalis JU1373	Insertions in unannotated regions (bp)	670799
C. inopinata	Insertions in unannotated regions (bp)	26021672
C. nigoni	Insertions in unannotated regions (bp)	11466867
C. sinica	Insertions in unannotated regions (bp)	2630752

Table 6. Insertion and Inversion/Transposition sizes for *Caenorhabditis* species and strains with assembled sequences contained in <100 contiguous pieces. Insertions and Inversions/Transpositions are calculated in the species genome coordinates.

	Insertion number (genes)	Mean size (Median) +/- sd	Insertion number (TEs)	Mean size (Median) +/- sd	Inversion/Transposition number (genes)	Mean size (Median) +/- sd	Inversion/Transposition number (TEs)	Mean size (Median) +/- sd
C. remanei PX506	52906	38 (3) +/- 166	49571	186 (7) +/- 498	1171	7148 (484) +/- 15121	1184	2672 (362) +/- 5996
C. remanei PX356	80509	30 (3) +/- 132	46695	140 (6) +/- 456	1046	2107 (265) +/- 6367	1170	2555 (593) +/- 6130
C. remanei PX439	76506	34 (3) +/- 162	44695	120 (5) +/- 398	1113	2083 (239) +/- 5489	1286	3009 (687) +/- 5909
C. latens	176044	29 (4) +/- 153	64298	139 (7) +/- 435	1000	260 (55) +/- 771	619	358 (88) +/- 734
C. briggsae	242671	33 (3) +/- 169	58192	108 (5) +/- 372	1028	295 (92) +/- 581	420	386 (126) +/- 862
C. elegans	354081	78 (5) +/- 292	35386	385 (10) +/- 840	958	117 (29) +/- 250	158	132 (48) +/- 255
C. tropicalis NIC58	18988	27.36 (3) +/- 278	4679	661 (4) +/- 2307	551	56999 (845) +/- 103358	140	13000 (260) +/- 42490
C. inopinata	224273	149 (5) +/- 536	90250	903 (322) +/- 1421	799	165 (62) +/- 334	501	161 (74) +/- 330
C. nigoni	214105	49 (3) +/- 496	62558	207 (5) +/- 1257	1284	171 (49) +/- 368	577	200 (87) +/- 330

Table 7. Deletion and Duplication sizes for *Caenorhabditis* species and strains with assembled sequences contained in <100 contiguous fragments. Deletions and Duplications are calculated in ancestor genome coordinates and can not be associated with annotated gene or TE features.

	Deletion number	Mean size (Median) +/- sd	Duplication number	Mean size +/- sd
C. remanei PX506	61380	6 (2) +/- 14	211249	85 (21) +/- 238
C. remanei PX356	154383	9 (2) +/- 41	140127	83 (22) +/- 217
C. remanei PX439	128950	9 (2) +/- 38	179330	88 (22) +/- 219
C. latens	85313	4 (2) +/- 9	304085	61 (17) +/- 165
C. briggsae	201676	4 (2) +/- 16	126817	67 (16) +/- 238
C. elegans	193488	4 (3) +/- 10	346466	32 (9) +/- 98
C. tropicalis NIC58	4052	6 (2) +/- 47	22736	104 (24) +/- 353
C. inopinata	141412	4 (3) +/- 18	341310	46 (11) +/- 123
C. nigoni	71457	8 (3) +/- 49	551878	38 (11) +/- 108

Table 8. *k*-mer analysis for the *de novo* assembled *C. remanei* PX356 and PX439 strains and *C. latens*. Potential false duplications would appear as Over Represented *k*-mers "OverRep" while incorrectly collapsed regions of the genome would appear as Under Represented *k*-mers "UnderRep". All 3 genome sequences show a low proportion of Over Represented *k*-mers.

	Total k-mers	Good	Error	OverRep	Low UnderRep	High UnderRep
C. remanei PX356	137,891,928	115,611,740	594,157	650,041	5,281,248	50,147
C. remanei PX439	145,723,210	131,088,130	198,160	291,686	1,505,584	174,467
C. latens	113,606,857	58,631,208	1,090,262	132,877	31,777,461	2,435,475

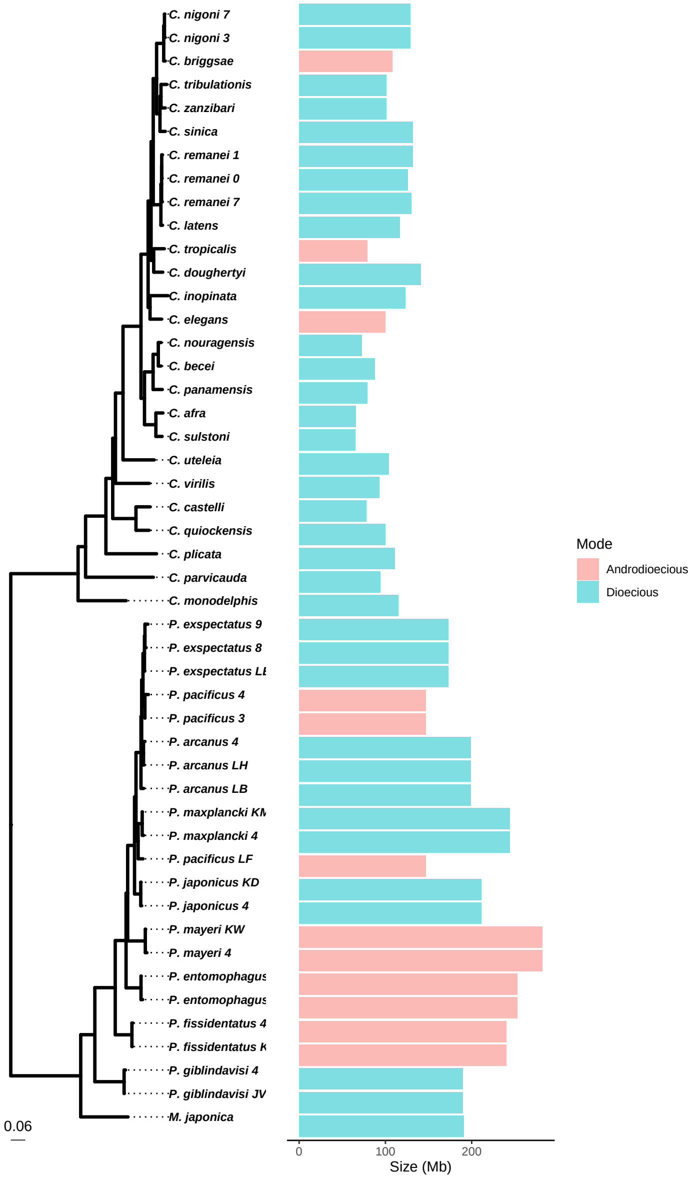

Fig. 1: Available genome size estimates for *Caenorhabditis* and *Pristionchus* species. Branch lengths are in terms of nucleotide substitutions and the scale bar is shown in the bottom left of the figure.

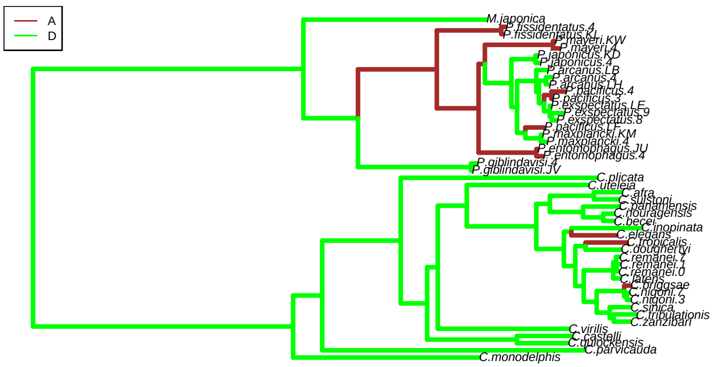

Fig. 2: The *Caenorhabditis* and *Pristionchus* phylogeny and trait values used for ancestral state reconstruction.

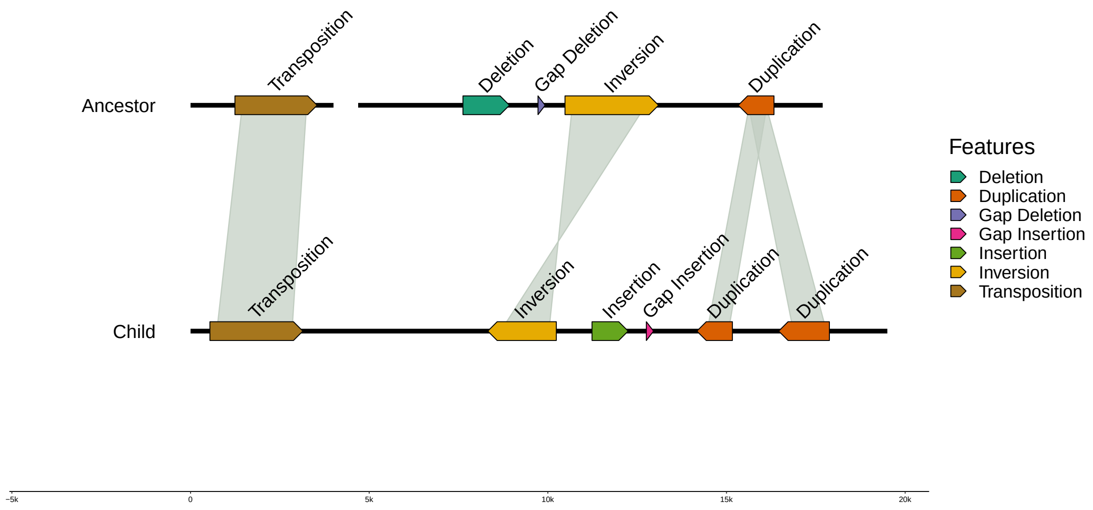

Fig. 3: Genome size changes from ancestor to child can happen by transposition (here shown as 'cut-and-paste', small (gap) and large deletions, small (gap) and large insertions, inversions and duplications. Within the ancestral genome reconstruction and mutation estimate protocols we used these can be of any size and do not need to contain full CDS or flanking sequences.

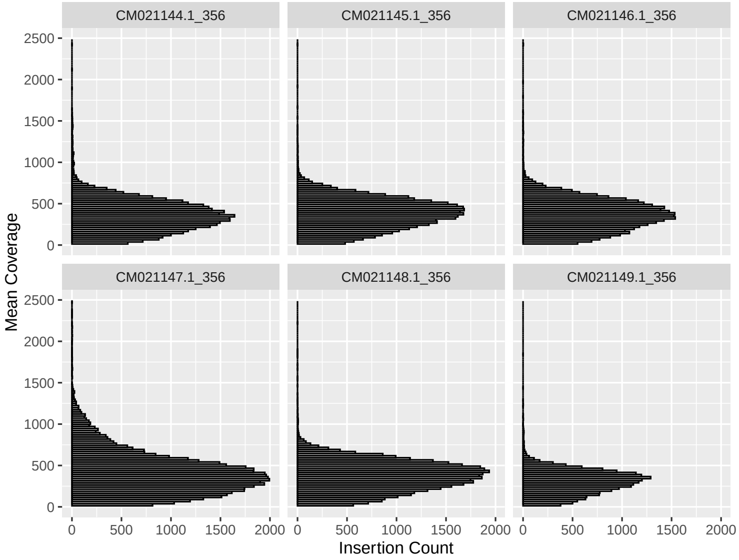

Fig. 4: DNA library coverage across the inserted regions of *C. remanei* PX356 shows little bias towards falsely expanded or duplicated sequences. Here, artefactual expansion would appear as regions of unusually low sequencing coverage.

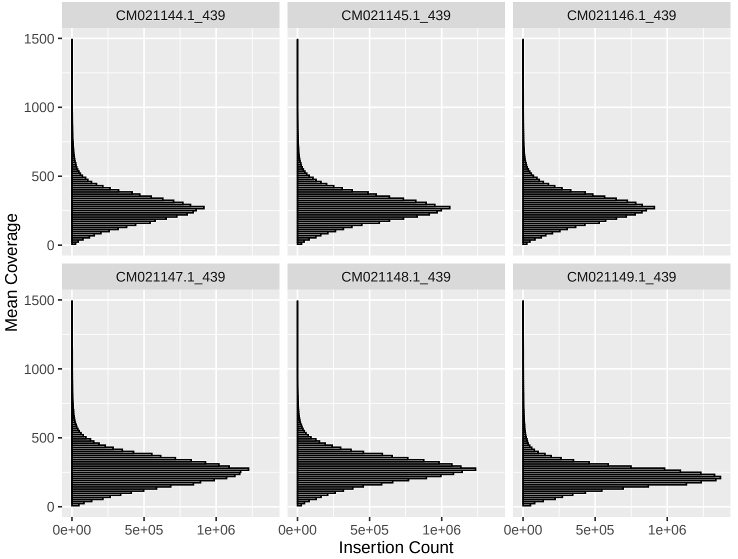

Fig. 5: DNA library coverage across the inserted regions of *C. remanei* PX439 shows little bias. Similar to the other library coverage profiles, false expansion would appear as biased regions of low sequencing coverage.

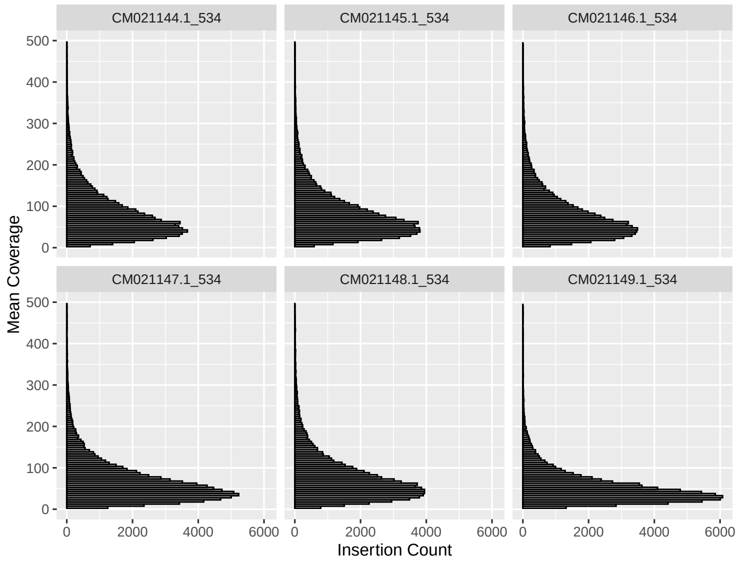

Fig. 6: DNA library coverage across the inserted regions of *C. latens* shows little bias. Systematic issues with false duplications and inappropriate expansions in the assembled sequence would appear as regions of excessively low sequencing coverage.

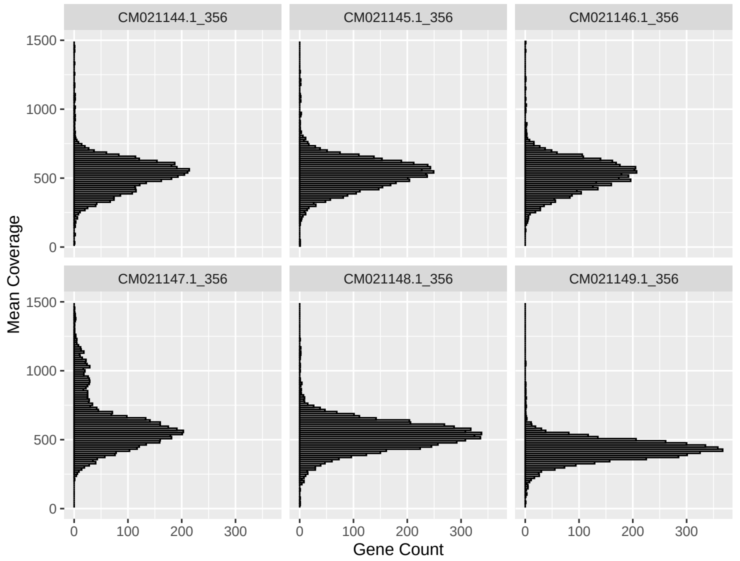

Fig. 7: DNA library coverage across the genic regions of *C. remanei* PX356 demonstrates little tendency towards artificial expansion of genic sequences. Instead, the coverage patterns suggest some genes have been inappropriately 'collapsed' in CM021147.1 (indicated by a region of roughly double the mean coverage at approximately 1000x).

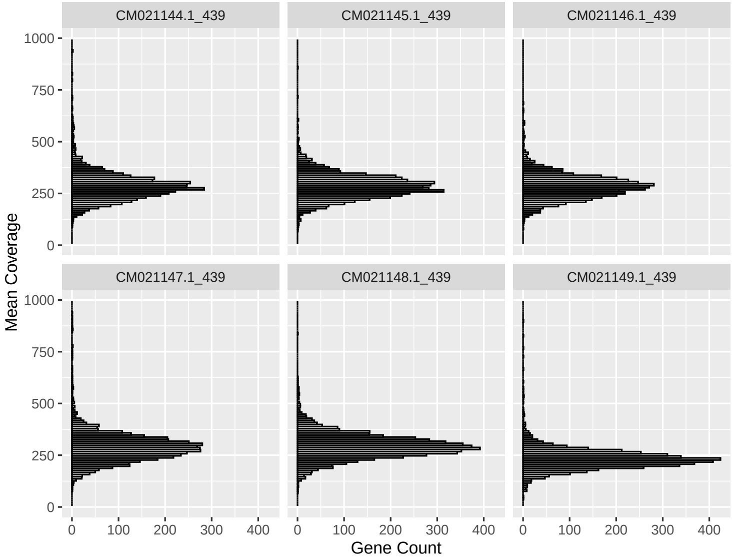

Fig. 8: DNA library coverage across the inserted regions of *C. remanei* PX439 shows little bias towards false duplications.

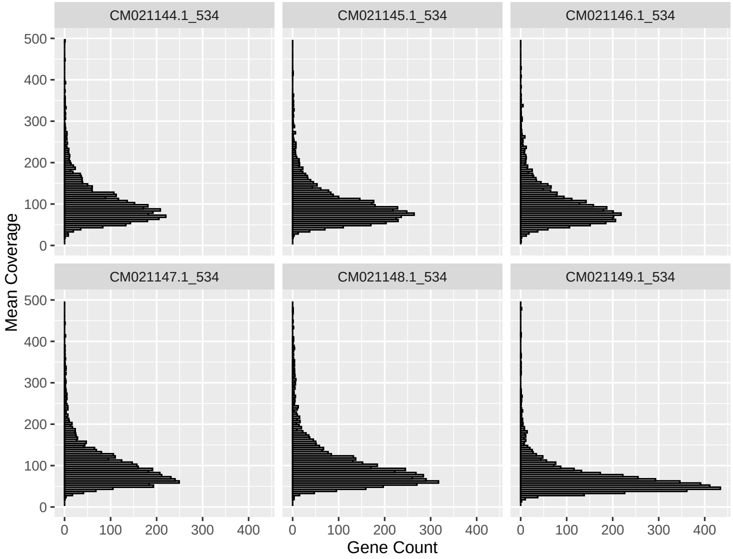

Fig. 9: DNA library coverage across the inserted regions of *C. latens* shows little bias towards falsely inflated sequences.

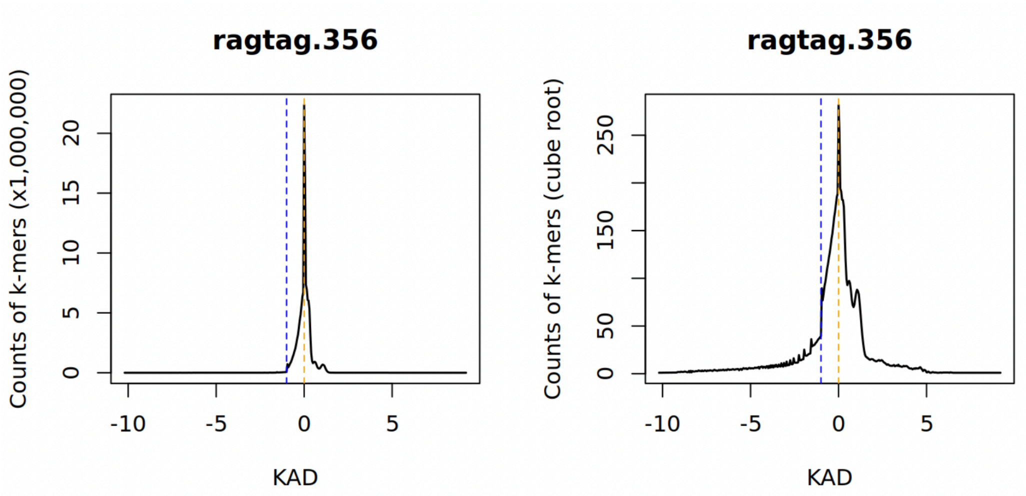

Fig. 10: Analysis of k-mer frequencies in the *C. remanei* PX356 genome sequences shows little tendency towards artefactual duplication or expansion (here, shown as k-mers with frequencies less than 2).The dotted blue line indicates k-mer frequencies of -1 and the dotted orange line indicates k-mer frequencies of 0.

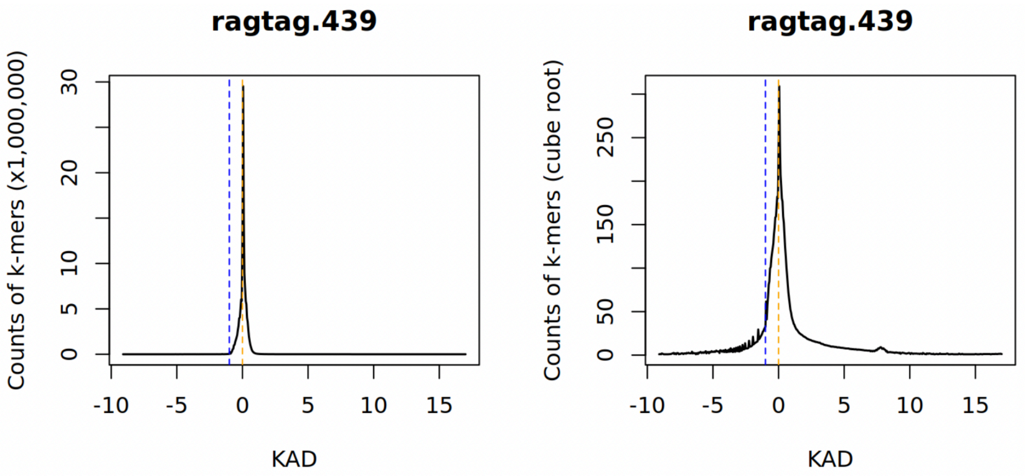

Fig. 11: Similar to the *C. remanei* PX356 genome sequences, the *C. remanei* PX439 genome sequences show very few *k-mers* with possible false duplication. The dotted blue line indicates k-mer frequencies of -1 and the dotted orange line indicates k-mer frequencies of 0.

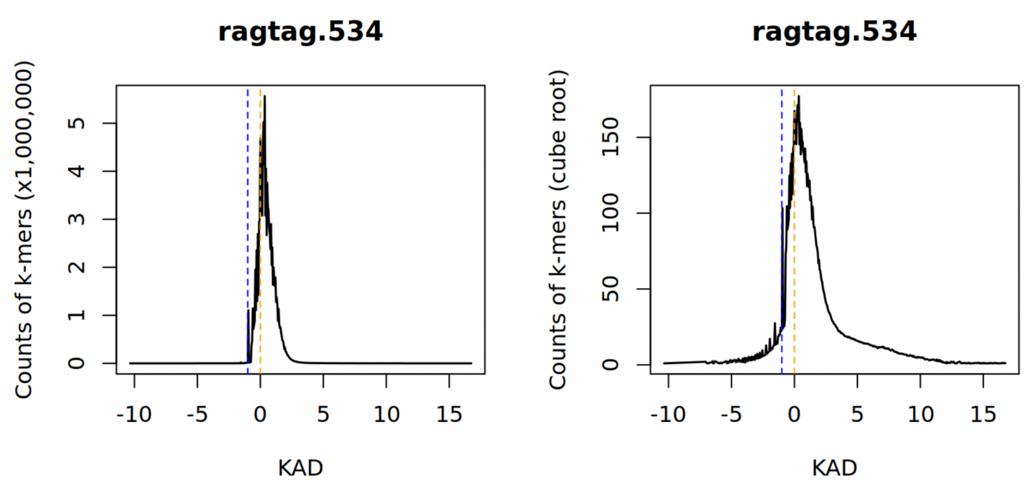

Fig. 12: The *C. latens* k-mer frequencies demonstrate little tendency towards falsely inflated or duplicated regions of the genome sequence. The dotted blue line indicates k-mer frequencies of -1 and the dotted orange line indicates k-mer frequencies of 0.

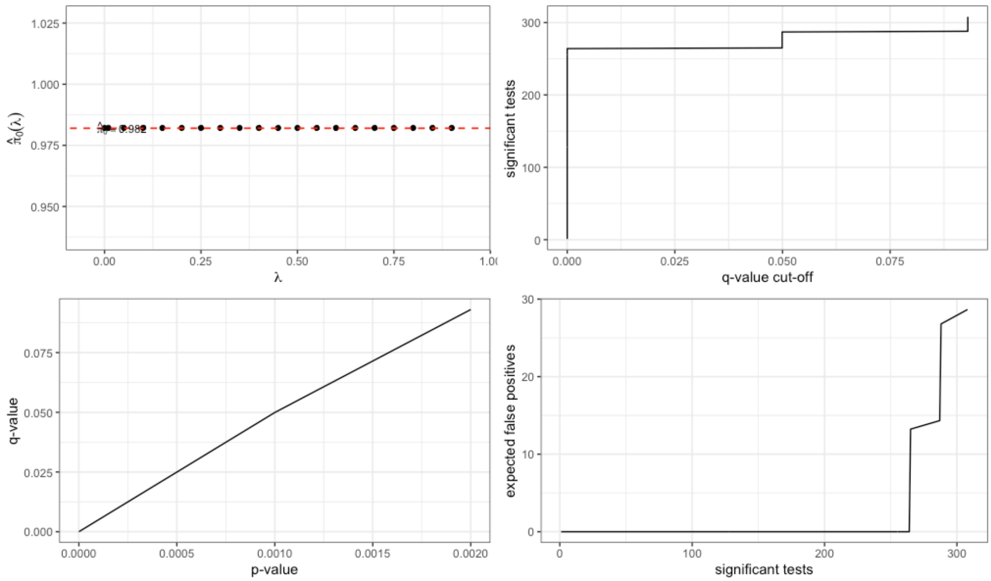

Fig. 13: The distribution of q-values, significance and expected false positives in the CAFE5 analyses.
